# Low-Processed Extracts from Peppermint Leaves (*Mentha* × *piperita* L.) as a Source of Polyphenols and Essential Oils: Evaluation of Green Solvents and Valorization of Post-Extraction Plant Material

**DOI:** 10.3390/molecules31071128

**Published:** 2026-03-29

**Authors:** Radosław Kowalski, Klaudia Kałwa, Artur Mazurek, Grażyna Kowalska

**Affiliations:** 1 Department of Analysis and Evaluation of Food Quality, University of Life Sciences in Lublin, 20-704 Lublin, Poland; artur.mazurek@up.lublin.pl; 2 The Institute of Human Nutrition Science and Agricultural, Department of Dietetics, The State University of Applied Sciences in Chełm, 22-100 Chełm, Poland; klaudia.kalwa91@gmail.com; 3 Department of Tourism and Recreation, University of Life Sciences in Lublin, 20-950 Lublin, Poland; grazyna.kowalska@up.lublin.pl

**Keywords:** peppermint, green solvents, low-processed extracts, infusion-like extraction, polyphenols, flavonoids, essential oil, secondary extraction, circular economy

## Abstract

This study examines a low-processed, food-grade extraction concept for peppermint leaves (*Mentha* × *piperita* L.) using solvents consistent with the principles of green chemistry and an infusion-like protocol. Primary extraction (2–30 min; 50–100 °C) was carried out using water, plasma-treated nanowater, a glycerol–water mixture (65%), an ethanol–water mixture (50%; at room temperature and at 50 °C), and rapeseed oil. To evaluate the potential use of biomass within a circular economy model, the residue remaining after the first extraction was subjected to secondary extraction under identical time–temperature conditions. Primary and secondary extracts were characterized in terms of total phenolic content (TPC), total flavonoid content (TFC), essential oil (EO) recovery, and antioxidant activity (DPPH and FRAP), and extraction yields were expressed relative to a 70% methanolic reference (TPC/TFC) and to the initial EO content in the plant material. Under the most favorable conditions (10 min; 100 °C; ethanol–water at 50 °C), the highest extraction yields of polar phytochemicals (TPC and TFC) were obtained with water and nanowater, whereas the ethanol–water mixture (50%) and rapeseed oil provided the greatest recovery of essential oil (up to complete depletion after the second extraction). Antioxidant activity showed a similar dependence on solvent type, with water and nanowater extracts exhibiting the highest DPPH/FRAP values. Importantly, secondary extraction contributed a substantial share of the total recovered bioactive compounds (often >30% of combined TPC/TFC), confirming that post-extraction residues remain a valuable raw material. The results support a practical, sequential strategy for designing peppermint extracts: aqueous and glycerol systems for phenolic-rich extracts, and ethanol and oil systems for essential-oil-enriched preparations, with secondary extraction enabling simple, low-energy biomass valorization.

## 1. Introduction

In the context of contemporary challenges, such as the need to reduce the use of chemical agents and the pursuit of more sustainable and environmentally friendly methods for processing plant raw materials, peppermint and its extracts are becoming an object of growing interest. Of particular importance is the production of low-processed plant extracts, which align with the principles of green chemistry and sustainable food production.

The term ‘low-processed herbal extracts’ has not yet been clearly defined in legal regulations nor firmly established in the scientific literature; however, it is increasingly used in the context of phytotherapy, dietetics, and supplement production as an opposite to highly purified or standardized extracts [1]. This concept refers to the general trend of minimal processing in the production of foods and plant-based preparations. Low-processed herbal extracts are obtained from plant materials using mild technological methods (e.g., maceration, infusions, decoctions, hydroalcoholic extraction), without intensive physicochemical processing (such as fractionation, chemical purification, standardization, or isolation of active compounds) [1]. In this study, “low-processed extracts” are operationally defined as end-use liquid extracts obtained directly after extraction and separation of plant material, without any downstream concentration (e.g., evaporation), solvent removal, purification/fractionation, or chemical standardization, i.e., the liquid phase after filtration/decantation was analyzed and considered the final product. They retain the full or nearly full spectrum of biologically active substances present in the plant, which enables the achievement of synergistic effects resulting from the combined action of bioactive constituents. Hereafter, for consistency, we use the term ‘low-processed extracts’ in the operational sense defined above.

The extraction procedure should not be complicated and should be characterized by low energy consumption as well as the use of green solvents that are both safe and environmentally friendly. In the present context, “safe” refers primarily to food-grade solvents acceptable for technological and consumer-oriented applications, whereas “environmentally friendly” refers to solvents and procedures associated with lower toxicity, reduced processing burden, and better compatibility with sustainable extraction concepts. Such solvents include glycerol, nanowater, vegetable oils, and water–ethanol mixtures, which not only exhibit high extraction efficiency but are also biodegradable and safe for consumers and the environment. Increasing attention is also being paid to the concept of using simple techniques inspired by the classical procedure for preparing tea infusions (PN-ISO 3103:1996) [2,3], which are easy to implement in practice and are regarded as low-energy because they require short extraction times, simple equipment, and no additional concentration or solvent-removal steps. In both industrial and laboratory practice, the literature is still dominated by water/ethanol systems and, in many cases, organic solvents, whereas food-grade glycerol–water extraction remains comparatively less explored despite its growing relevance within green-chemistry frameworks [4,5,6,7,8,9,10]. Unlike more intensified green extraction techniques, such as ultrasound-assisted or microwave-assisted extraction [6,9,10], the approach used in this study was intentionally based on a simple infusion-like protocol employing food-grade solvents and minimal technological requirements. Although assisted techniques may improve extraction efficiency or shorten processing time, the present concept emphasizes direct applicability, low processing intensity, and the possibility of obtaining end-use liquid extracts without downstream treatment.

Among plant species that provide key ingredients for the production of herbal dietary supplements with documented health-promoting properties, peppermint (*Mentha* × *piperita* L.) occupies a particularly important position. This plant is characterized by a rich phytochemical profile and a broad spectrum of biological activity. The literature confirms, among others, its antioxidant, antidiabetic, immunomodulatory, antiparasitic, cardioprotective, hypolipidemic, and spasmolytic effects [11,12,13,14,15]. The bioactivity of peppermint is mainly associated with the presence of polyphenols (rosmarinic acid, caffeic acid, chlorogenic acid, flavonoids) as well as essential oil, whose main constituents are menthol and menthone [16].

In the present study, low-processed extracts from peppermint leaves were obtained using readily available solvents, i.e., water, nanowater, a glycerol–water solution, an ethanol–water solution, and rapeseed oil, under variable temperature and time conditions, applying a procedure corresponding to the preparation of classical aqueous infusions. This study was designed to assess whether such an approach could improve the recovery of bioactive compounds while also fitting the assumptions of circular economy and waste-biomass valorization [17,18]. The main objective of this study was to evaluate the selective recovery of polyphenols, flavonoids, antioxidant activity, and essential oil-related fractions from peppermint leaves using low-processed, food-grade extraction systems under infusion-like conditions. It was hypothesized that solvent polarity and phase affinity would significantly affect extraction selectivity, with aqueous and glycerol-based systems favoring the recovery of polar constituents, and ethanol- or oil-based systems favoring the recovery of the volatile/lipophilic fraction. A further objective was to assess whether post-extraction plant material could still serve as a valuable source of bioactive compounds in a secondary extraction step. The current relevance of this work arises from the growing interest in food-grade, low-processed extraction systems compatible with sustainable production, whereas its novelty lies in the combined evaluation of solvent-dependent selectivity and secondary extraction as a route for valorizing post-extraction peppermint biomass.

## 2. Results and Discussion

### 2.1. Determination of Boundary Conditions for Extraction Time

**Figure 1:**
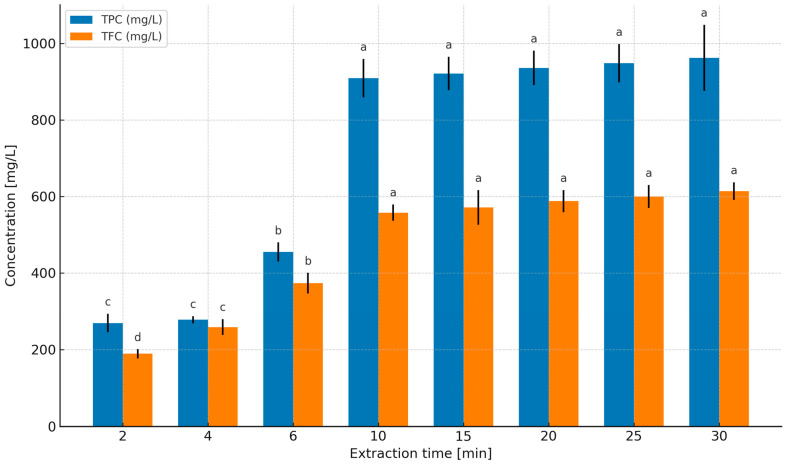
TPC and TFC in aqueous extracts obtained using extended extraction times of 1–30 min (at an initial extraction temperature of 100 °C). Error bars represent standard deviation (SD) from three independent replicates. Different letters (a, b, c, etc.) indicate statistically significant differences at *p* < 0.05.

In order to determine the optimal extraction time, reference was made to the PN-ISO 3103 standard [3], which standardizes the preparation of tea infusions (6 min) for sensory evaluation. In the present study, however, the focus was placed on the chemical parameters of the extracts (TPC—total phenolic content, TFC—total flavonoid content), without organoleptic assessment. In aqueous extracts, both TPC and TFC increased with prolonged extraction time—from 268.99 to 962.10 mg GAE/L (GAE—gallic acid equivalents, TPC) and from 189.02 to 613.76 mg ECE/L (ECE—epicatechin equivalents, TFC) within the 2–30 min range (Figure 1). The largest increase in concentrations was observed between 2 and 10 min (TPC: 268.99 → 909.10 mg GAE/L; TFC: 189.02 → 557.59 mg ECE/L), whereas further extension of the extraction time to 30 min resulted in disproportionately small gains (TPC: 909.10 → 962.10 mg GAE/L; TFC: 557.59 → 613.76 mg ECE/L). From the perspective of process economics (energy/time), maintaining extraction times longer than 10 min is therefore inefficient relative to the increase in the concentration of bioactive compounds.

**Figure 2:**
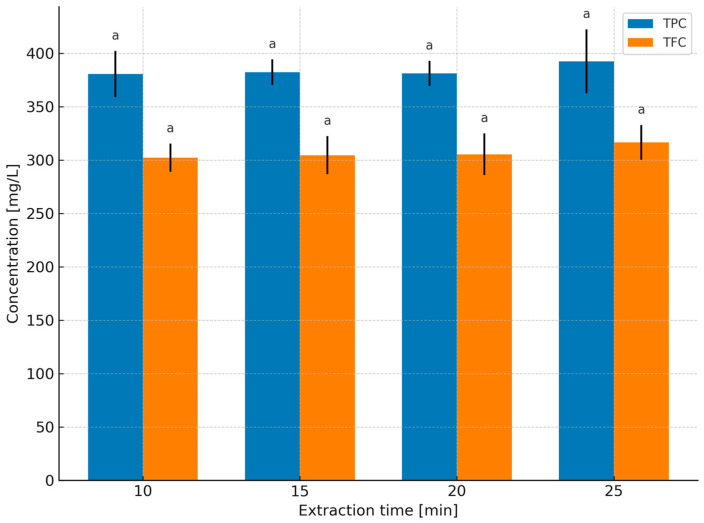
TPC and TFC in 65% glycerol–water extracts obtained using extended extraction times of 10–25 min (at an initial extraction temperature of 100 °C). Error bars represent standard deviation (SD) from three independent replicates. Different letters (a) indicate statistically significant differences at *p* < 0.05.

In the 65% glycerol–water system (Figure 2), no significant increases were observed after 10 min. Within the 10–25 min range, TPC remained statistically unchanged, varying between 380.50 and 392.41 mg GAE/L, while TFC ranged from 302.12 to 316.40 mg ECE/L. These data indicate the occurrence of a kinetic plateau after approximately 10 min, which justifies the selection of 2–10 min as the target extraction time window in further comparative solvent experiments.

Numerous literature reports have demonstrated that both extraction time and temperature in tea and herbal infusions systematically affect the levels of bioactive compounds as well as their release kinetics. Labbé et al. [19], using tea as a model, showed that the extraction kinetics of major phenolic constituents are not uniform and depend on both extraction time and temperature. Their results illustrate that different classes of compounds display distinct mass-transfer and solubility profiles, leading to divergence in kinetic optima.

Observations reported by Dmowski et al. [20] and Ramalho et al. [21] confirm that the first several minutes of infusion provide the highest relative increase in phenolic compounds, supporting the conclusion that a rapid diffusion phase dominates the initial stage of the process. In turn, Kelebek [22], comparing 80 and 100 °C at 3, 6, and 10 min, demonstrated that increasing temperature shifts the entire concentration range of flavan-3-ols toward higher values (e.g., epicatechin increases with both temperature and time), which from a practical standpoint confirms the usefulness of short, high-temperature infusions for phenolic fractions.

Studies [23,24,25,26,27] also emphasize a second, equally important aspect: the balance between extraction benefits and heating costs. Numerous experiments have shown that increasing temperature enhances both solubility and diffusion coefficients, thereby accelerating extraction [23,24,25]. At the same time, excessively high temperatures and/or prolonged extraction times may intensify the degradation of certain phenolic compounds [23,24,26]. Spigno and De Faveri [26] reported higher phenolic yields at 60 °C compared with 28 °C; however, in a subsequent study, Spigno et al. [27] identified 45 °C as a compromise temperature that maintains high concentrations while reducing the risk of degradation and lowering energy costs, recommending that energy input at the extraction stage be evaluated in the context of total process costs [27]. In this regard, the present observations for peppermint—rapid increases up to 6–10 min followed by plateau formation in aqueous and glycerol-based systems—fit well with the “fast diffusion phase → diminishing marginal returns” mechanism described in [20,21,22,26,27].

With respect to extraction time, the literature does not indicate a single universal optimum. Some authors apply short extraction times of 5–30 min [24,25,28], which is consistent with infusion-type processes and lower operational costs, whereas others use much longer extraction periods [23,26,29,30,31]. These differences do not contradict one another but rather reflect varying objectives and constraints, including raw material characteristics (species, degree of comminution), solvent properties (polar vs. less polar, viscosity), target compound classes (e.g., phenolics vs. volatile fractions), and acceptable energy expenditure [23,24,25,29,30,31].

### 2.2. Assessment of the Suitability of Green Solvents for the Extraction of Active Compounds—Primary Extraction

**Figure 3:**
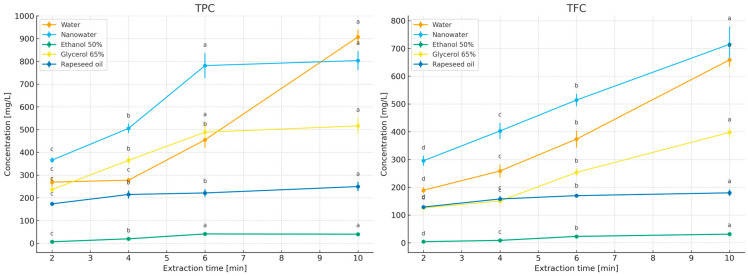
TPC and TFC in primary extracts (water, nanowater, 50% ethanol, 65% glycerol, and rapeseed oil) as a function of extraction time (initial extraction temperature 100 °C; 50% ethanol—50 °C). Error bars represent standard deviation (SD) from three independent replicates. Different letters (a, b, c, etc.) indicate statistically significant differences at *p* < 0.05.

**Figure 4:**
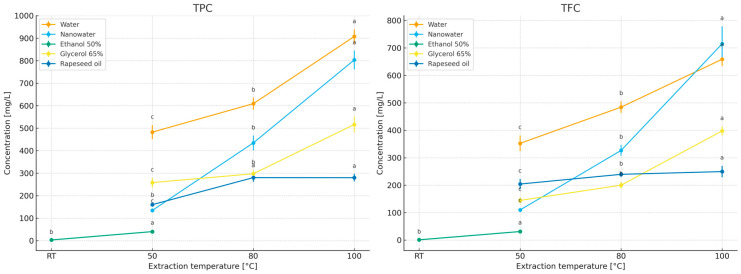
TPC and TFC in primary extracts (water, nanowater, 50% ethanol, 65% glycerol, and rapeseed oil) as a function of extraction temperature (extraction time 10 min). Error bars represent standard deviation (SD) from three independent replicates. Different letters (a, b, c) indicate statistically significant differences at *p* < 0.05.

The study compared the effects of extraction time (2–10 min; Figure 3) and temperature (50–100 °C; for 50% ethanol—room temperature and 50 °C; Figure 4) on the contents of polyphenols (TPC) and flavonoids (TFC) in primary peppermint extracts obtained using water, nanowater, 65% glycerol, 50% ethanol, and rapeseed oil. Because the tested solvents differed markedly in polarity, viscosity, and technological constraints related to thermal handling, especially in the case of the ethanol–water system, the 50% ethanol variant was evaluated at room temperature and 50 °C rather than under near-boiling conditions, and it was included primarily as a food-grade hydroalcoholic reference relevant to practical extract applications [32,33]. In addition, in the section devoted to essential oils (EO), the behavior of the volatile fraction as a function of time and temperature was compared, allowing the identification of different solvent optima for polar compounds (TPC/TFC) and lipophilic compounds (EO).

Water served as the reference solvent. With increasing extraction time from 2 to 10 min, TPC increased from 268.99 to 907.50 mg GAE/L, and TFC from 189.02 to 658.74 mg ECE/L (Figure 4), confirming that under short infusion conditions a rapid extraction of readily releasable fractions from leaf tissue occurs (diffusion combined with tissue swelling; [19,20,21]). Increasing temperature further enhanced this effect, with the highest levels obtained at 100 °C/10 min (Figure 4), consistent with observations for classical infusions where intensified mass transfer and increased solubility of phenolics at elevated temperatures dominate over potential degradation during short extraction times [22,23,24,25,26,27,28,29,30,31]. After 10 min, the curves reached a plateau (Figure 2), which from the perspective of process economics (energy/time) supports maintaining extraction within a short 2–10 min window.

Tahirovic et al. [34] reported slightly higher polyphenol contents, showing that the polyphenol level in an aqueous peppermint extract was 137.1 mg GAE/100 mL, corresponding to 1371 mg GAE/L. In contrast, Cho et al. [35] obtained more than two-fold higher concentrations—2.26 mg GAE/mL (2260 mg GAE/L). Significantly lower polyphenol concentrations in peppermint infusions than those obtained in the present study were reported by Farcas et al. [36] and Atoui et al. [37], i.e., 39.3 mg GAE/100 mL (393 mg GAE/L) and 106 mg GAE/240 mL (441.67 mg GAE/L), respectively—values more than two-fold lower than those reported here.

The results indicate a clear increasing trend in polyphenol and flavonoid concentrations with increasing extraction temperature. The positive effect of temperature on phenolic extraction efficiency has also been reported in other studies [27,38,39,40,41]. These authors attribute the increased yields to both enhanced solubility of phenolic compounds in the solvent and intensified diffusion from the interior of plant cells into the extraction medium. It has been reported that, in some extraction systems, the most favorable temperature range is 50–70 °C, whereas at 90–100 °C a decrease in extraction efficiency may occur due to thermal degradation of polyphenolic molecules [40]. However, such effects depend strongly on extraction time, plant matrix, and solvent system; in the present study, the short extraction time (10 min) most likely allowed the enhancing effect of temperature on mass transfer and solubility to dominate over possible thermal losses.

Nanowater, when used as an extractant, did not outperform water in terms of TPC/TFC concentrations, despite similar time- and temperature-dependent trends (Figure 3 and Figure 4). To the best of our knowledge, published data on the use of nanowater for extracting bioactive compounds from herbs remain limited, and the available information is based mainly on a small number of exploratory studies and broader discussions of green solvent systems [1]. Nanowater is produced by removing water clustering in a low-temperature plasma reactor, which alters its structure and confers specific physicochemical properties, including enhanced availability of soil nutrients to plants [42]. According to the characterization discussed in our previous book chapter [1], the commercial nanowater applied in the present study has mineralization comparable to that of typical tap water. According to the manufacturer, tap water is used as the raw material and subsequently subjected to plasma treatment. In practical terms, the manufacturer describes this product as a ROS-enriched, plasma-modified water (including singlet oxygen), which may transiently alter redox properties and interfacial behavior during contact with plant tissue. Such changes could influence extraction indirectly via modified wetting/penetration of the leaf matrix and/or by promoting oxidation of redox-sensitive constituents during extraction and assay development. However, because we did not perform a full physicochemical characterization of the specific nanowater batch used (e.g., ORP, conductivity, dissolved oxygen/ROS markers), mechanistic interpretation should be treated as tentative (see Limitations). It can be hypothesized that the presence of cations (especially Ca^2+^ and Mg^2+^) may have modified colorimetric responses in spectrophotometric assays (Folin–Ciocalteu, AlCl_3_) through complex formation with phenolics or activation of functional groups, thereby affecting measured (though not necessarily actual) concentrations and antioxidant activity [43,44,45]. Such ionic interference effects have previously been reported for phenolics in aqueous systems [43], which may explain the several-to-ten percent differences observed here between water and nanowater despite similar extraction kinetics [45,46]. EO content was not evaluated separately in nanowater, as preliminary experiments did not indicate advantages over water (comparable values in short infusions), and phase separation and solubility of volatile fractions in purely aqueous environments are limited [47]. Additionally, economic considerations are relevant, as nanowater is several tens of times more expensive than water.

**Figure 5:**
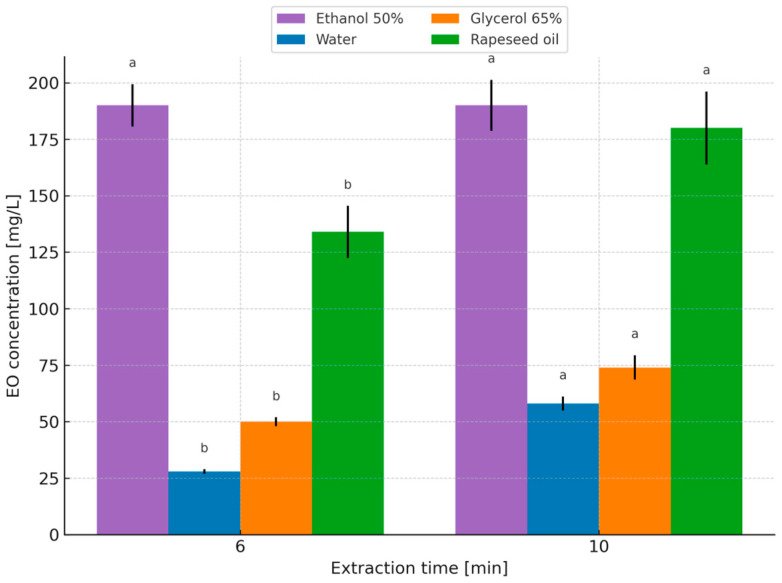
Essential oil content in extracts (50% ethanol, water, 65% glycerol, and rapeseed oil) as a function of extraction time (initial extraction temperature 100 °C). Error bars represent standard deviation (SD) from three independent replicates. Different letters (a, b) indicate statistically significant differences at *p* < 0.05.

For 50% ethanol extraction, the contents of bioactive compounds increased with higher initial extraction temperatures and reached maximum levels at 10 min. Under short, mild infusion conditions, 50% ethanol consistently yielded the lowest TPC/TFC concentrations (e.g., 2–10 min: TPC 6.84 → 40.20 mg GAE/L; TFC 3.94 → 31.02 mg ECE/L—Figure 4; RT–50 °C—Figure 5). This outcome reflects a polarity mismatch with the main peppermint phenolics (rosmarinic acid and derivatives) and the fact that short extraction times in hydroalcoholic systems do not allow sufficient tissue swelling compared with water. Thus, the results suggest that solvent polarity strongly influenced the extraction of phenolics and flavonoids in this system: highly polar aqueous media promoted the recovery of peppermint phenolics during short extraction, whereas less polar or more viscous systems reduced the efficiency of releasing these constituents into the liquid phase. In the present study, methanol was not treated as an alternative food-grade extraction system, but as an exhaustive reference solvent used to establish the relative recovery of TPC and TFC under low-processed extraction conditions. Ethanol and methanol are both widely used in exhaustive extraction protocols aimed at recovering broad groups of bioactive compounds. Literature data do not indicate one universal superiority of either solvent, as the extraction efficiency depends on the target compound group and extraction conditions [31,41,48,49,50,51,52,53,54]. In this context, ethanol was included in the present study as a practical food-grade hydroalcoholic system, whereas methanol served exclusively as the exhaustive reference solvent.

An important factor in designing an appropriate extraction system is selecting a solvent composition suited to the target group of compounds, most commonly in combination with water. Polar solvents such as methanol and ethanol, either pure or in aqueous mixtures, are frequently recommended for extracting phenolic antioxidants from plant materials [55,56]. However, in most cases a single solvent is insufficient to achieve maximum antioxidant yields across diverse plant matrices [57]. For this reason, the aim of the present study was not to identify one universally best solvent, but rather to compare the selective performance of several food-grade extraction systems differing in polarity and phase affinity. Anwar et al. [58] showed that for single-step extraction of barley polyphenols, 80% methanol was more effective than 100% methanol.

The rapeseed oil system yielded low TPC/TFC concentrations (limited solubility of polar fractions in the lipid phase and longer wetting times of the plant material): TPC 173.52–249.49 mg GAE/L and TFC 128.67–179.80 mg ECE/L. When oils are used as extractants, it should be considered that they are rich in mono- and polyunsaturated fatty acids, including essential fatty acids (EFAs) [59], and are therefore highly susceptible to oxidation, requiring protection from light, heat, oxygen, and water during storage [60]. Studies by Kozłowska and Żontała [61], demonstrated that the addition of plant extracts to sunflower oil improves its oxidative stability, supporting the potential use of oils as solvents for extracting bioactive compounds from herbs.

No statistically significant differences were observed in TPCs and TFCs between extracts obtained with 65% and 50% aqueous glycerol (Table 1): 520.36 mg GAE/L and 401.25 mg ECE/L (50% glycerol) versus 516.33 mg GAE/L and 397.62 mg ECE/L (65% glycerol). With 65% glycerol, TPC/TFC increased with extraction time (Figure 4), but absolute concentrations were lower than those obtained with water during short infusions, reflecting higher viscosity, reduced diffusivity, and subtle differences in solubility parameters relative to typical peppermint phenolics. Increasing temperature (Figure 4) markedly “unlocked” extraction kinetics (reduced viscosity, increased solubility and mass-transfer rates), such that at 100 °C/10 min the 65% glycerol system yielded the highest TPC and TFC levels in this comparison. Importantly, Table 1 shows that at 100 °C/10 min there were no significant differences between 50% and 65% glycerol extracts, suggesting that solvent concentration can be selected based on operational criteria (viscosity, filtration ease, cost, extract sensory properties) rather than expected yield gains [5,62,63].

In food production practice, glycerol mixtures offer an additional stabilizing benefit (reduced water activity and antioxidant protection), which may compensate for slightly lower short-time yields compared with water [62,64]. Glycerol is a biocompatible solvent with higher viscosity than water, suitable for food applications, characterized by low cost, non-toxicity, and lower polarity than water; in aqueous mixtures it modifies the dielectric constant and can enhance extraction efficiency [7,8,65]. A drawback is its elevated viscosity, which may cause technological challenges, e.g., during filtration [5]. Nevertheless, the proposed use of glycerol for extracting bioactive substances from plant materials shows substantial industrial potential, particularly within green, environmentally friendly strategies for biomass utilization—especially food-processing residues—to produce high-value products such as natural food antioxidants, nutraceuticals, pharmaceuticals, and cosmetic ingredients. The TPC and TFC levels obtained in the present study for glycerol–water systems fit into the general trend described in the literature, according to which mixtures of glycerol with water may effectively extract phenolic compounds, although their efficiency depends strongly on the viscosity of the system, temperature, and the contact time with the plant material. In comparison with water, short-term extraction with glycerol gave lower absolute concentrations here, but these differences decreased with increasing temperature, which remains consistent with the observations of other authors concerning heated glycerol–water systems [5,7,62,63,64,65]. This means that, when interpreting the results for glycerol, not only should the composition of the solvent itself be taken into account, but also the mass-transfer limitations characteristic of more viscous systems.

**Figure 6:**
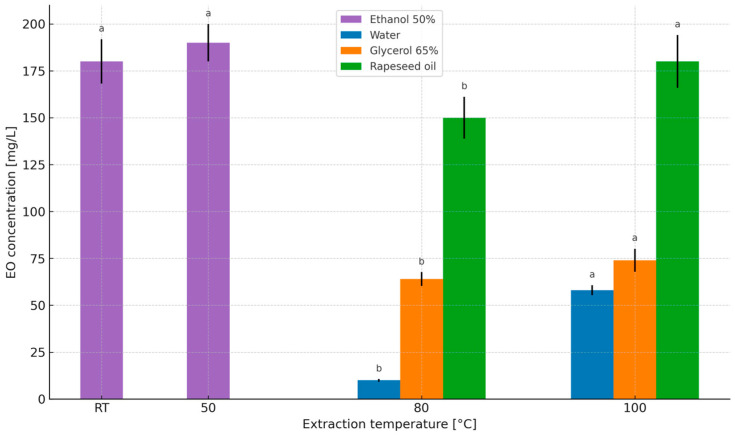
Essential oil content in extracts (50% ethanol, water, 65% glycerol, and rapeseed oil) as a function of extraction temperature (extraction time 10 min). Error bars represent standard deviation (SD) from three independent replicates. Different letters (a, b) indicate statistically significant differences at *p* < 0.05.

The concentrations of essential oils in herbal extracts (water, nanowater, 50% ethanol, 65% glycerol, and rapeseed oil) under the applied time and temperature variants are presented in Figure 5 and Figure 6.

The essential oil concentration in the prepared peppermint extracts increased proportionally with both extraction time and temperature, ranging from 10 mg/L (80 °C, 10 min) to 58 mg/L (100 °C, 10 min). Despite numerous studies assessing bioactive compounds in aqueous extracts, the influence of extraction conditions on essential oil content in such herbal infusions has rarely been addressed, possibly due to analytical complexity. In the present study, essential oil concentration was estimated using a calculation-based approach assuming no oil losses [47], enabling comparison of extraction efficiencies across solvent systems. Wyrostek et al. [47] reported that extending aqueous extraction time from 5 to 10 min increased essential oil concentrations in herbal infusions prepared from sage leaves, chamomile flower heads, lavender flowers, caraway fruits, fennel fruits, and peppermint leaves—findings consistent with the present results.

In 50% ethanol–water extracts of peppermint leaves, essential oil concentrations ranged from 180 mg/L (10 min, room temperature) to 190 mg/L (6 and 10 min at 50 °C). No literature reports were found addressing essential oil content in hydroalcoholic extracts under comparable conditions, highlighting the novelty of this aspect of the study. For rapeseed oil extracts, essential oil concentrations ranged from 134 mg/L (6 min at 100 °C) to 180 mg/L (10 min at 100 °C). One of the oldest aroma-extraction methods based on adsorption by fats is enfleurage, which exploits the high affinity of fats for aromatic compounds. The success of enfleurage depends largely on the quality of the fat base, which should be nearly odorless and possess appropriate consistency [66].

Similarly to aqueous extracts, essential oil concentrations in glycerol–water extracts of peppermint leaves depended on both extraction time and temperature. The highest oil concentration was 74 mg/L (100 °C, 10 min), while the lowest was 50 mg/L (100 °C, 6 min). Extraction using glycerol has been infrequently investigated for isolating bioactive compounds from herbs, and no studies were found evaluating essential oil content in glycerol or glycerol–water extracts under comparable conditions. Therefore, the present results provide a novel contribution, albeit difficult to benchmark directly against existing literature. Overall, extending extraction time and increasing temperature resulted in higher essential oil concentrations, consistent with previous findings for herbal infusions [47].

### 2.3. Secondary Extraction

**Figure 7:**
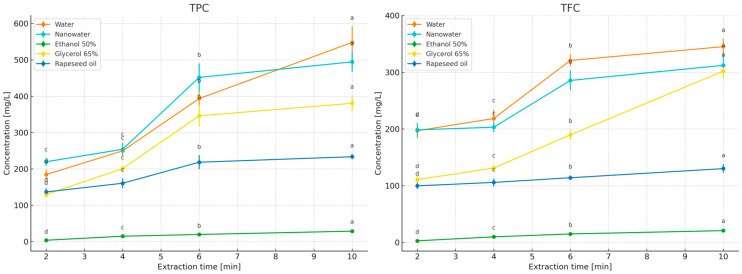
TPC and TFC in secondary extracts (water, nanowater, 50% ethanol, 65% glycerol, and rapeseed oil) as a function of extraction time (initial extraction temperature 100 °C). Different letters (a, b, c, etc.) indicate statistically significant differences at *p* < 0.05.

**Figure 8:**
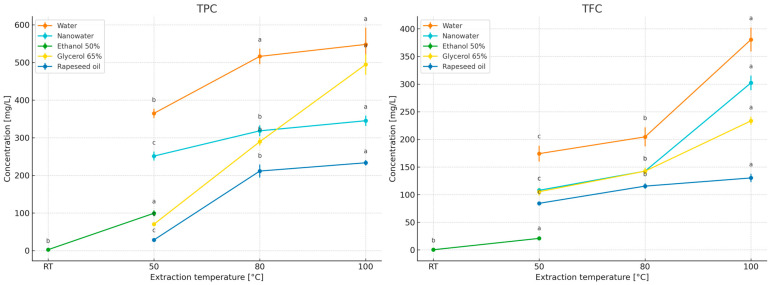
TPC and TFC in secondary extracts (water, nanowater, 50% ethanol, 65% glycerol, and rapeseed oil) as a function of extraction temperature at 10 min. Error bars represent standard deviation (SD) from three independent replicates. Means denoted by the same letters (a, b, c) do not differ significantly at the 5% level (α = 0.05).

Figure 7 and Figure 8 present the results for TPC and TFC concentrations in extracts obtained from the post-extraction material (so-called secondary extracts). The extraction process was performed using the same time–temperature parameters as those applied for extracts obtained in the first extraction step (primary extraction).

For the secondary extracts obtained from peppermint leaves recovered after the initial extraction, it was demonstrated that the post-extraction material still contained active compounds that could be released during a subsequent extraction step. This finding indicates that the time–temperature parameters applied for the primary extracts allow the attainment of optimal concentrations at 100 °C and 10 min, where an apparent concentration equilibrium is established between the solvent phase and the plant matrix; therefore, extending the extraction time is not effective for obtaining more concentrated primary extracts. In contrast, conducting a second extraction step (secondary extraction) enables recovery of the remaining fraction of active constituents. In the secondary extraction, similar relationships between extract concentration and the initial temperature of the extraction medium as well as extraction time were observed as in the primary extraction. The following maximum TPC concentrations were recorded: up to 547.00 mg GAE/L (water), up to 380.50 mg GAE/L (water–glycerol 65%), up to 494.52 mg GAE/L (nanowater), up to 28.50 mg GAE/L (ethanol–water 50%), and up to 233.46 mg GAE/L (rapeseed oil). Analogously to the primary extraction, TFC increased with prolonged extraction time. The following maximum total flavonoid concentrations were observed in peppermint leaf extracts: up to 345.20 mg ECE/L (water), up to 302.12 mg ECE/L (water–glycerol 65%), up to 312.29 mg ECE/L (nanowater), up to 20.85 mg ECE/L (ethanol–water 50%), and up to 130.18 mg ECE/L (rapeseed oil). Thus, in the case of TPC and TFC, secondary extraction primarily changed the quantitative recovery of the analyzed fractions, whereas for the essential-oil fraction a further section of the manuscript (Section 2.7) demonstrates that primary and secondary extraction also led to qualitative and quantitative differences in composition.

At present, a major challenge for many agri-food and industrial plants is the generation of large amounts of waste, which is often disposed of on agricultural land, posing a serious environmental risk [67]. However, as shown by the results presented in this study (Figure 7 and Figure 8), post-extraction plant residues contain substantial amounts of biologically active compounds with documented health-promoting properties [68,69,70,71,72]. Recovering phenolic compounds from post-extraction waste would provide dual benefits for both the environment and the pharmaceutical sector. Active substances are most commonly obtained from unprocessed plant raw materials; nevertheless, the possibility of obtaining bioactive compounds from plant residues remaining after industrial processing is also of interest, as are by-products generated during household food preparation. Such recovery pathways align well with the increasingly prominent concept of green chemistry. Rational management of natural materials is therefore desirable so that all components can be appropriately valorized, for example in the production of foods, medicines, dietary supplements, or cosmetics.

Wang et al. [73] reported that each subsequent extraction (secondary extraction) significantly reduced the contents of catechins and phenolic compounds in the resulting infusions. Secondary extraction can effectively release bioactive constituents, enabling the reuse of herbal residues remaining after the preparation of, for example, infusions. Coffee, tea, and herbs are regarded as some of the richest sources of bioactive compounds, containing substantial amounts of antioxidants and phenolic substances; therefore, residues from their processing may still contain considerable quantities of natural antioxidants [74]. Abdeltaif et al. [75] investigated polyphenol and flavonoid contents in primary and secondary extracts of coffee and black tea. They demonstrated that both coffee and tea contained significant amounts of polyphenolic compounds. The TPC in black tea after the first and second extraction was 187.50 mg GAE/g and 152.87 mg GAE/g, respectively, while in coffee it was 104.3 mg GAE/g and 97.87 mg GAE/g. Similarly, in the study by Kobus-Cisowska et al. [76], three successive extractions were performed using yellow and green Ginkgo biloba leaves, showing pronounced differences in phenolic content across consecutive extracts. Consistent with the present study, the total polyphenol content was highest after the first extraction and significantly lower in the second and third extracts, irrespective of the solvent used. For example, in the case of an aqueous extract of green tea leaves, polyphenol content in three successive extracts was 56.68 mg/g > 4.28 mg/g > 0.93 mg/g [76]. An analogous trend was observed for ethanol extraction: 204.50 mg/g > 6.91 mg/g > 1.79 mg/g [76]. Fecka and Turek [77] also demonstrated the effectiveness of secondary extraction for peppermint leaves, reporting polyphenol contents of 8.9 mg GAE/g (second extraction) and 1.7 mg GAE/g (third extraction). Wyrostek et al. [47] likewise found that secondary extracts from sage, lavender, caraway, fennel, chamomile, and peppermint exhibited significant levels of biologically active compounds from the investigated chemical groups.

**Figure 9:**
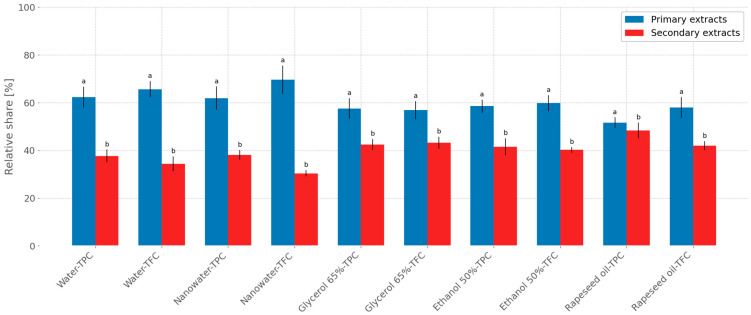
Relative percentage contribution of the analyzed active compounds in the total TPC and TFC fractions for primary and secondary extracts obtained under the most favorable extraction conditions, i.e., 10 min at 100 °C for water, nanowater, 65% glycerol, and rapeseed oil, and 10 min at 50 °C for the 50% ethanol system. Bars are arranged in paired form for each solvent system to facilitate comparison between primary and secondary extracts. Error bars represent standard deviation (SD) from three independent replicates. Different letters (a, b) indicate statistically significant differences at *p* < 0.05.

Figure 9 illustrates the percentage contribution of the analyzed active compounds in the total fraction (relative to TPC or TFC) for primary and secondary extracts, assuming that the combined TPC or TFC in both extracts represents 100%.

For aqueous extraction of peppermint leaves (Figure 9), the relative contribution of TPC recovered in the secondary extraction step, with respect to the total extract fraction (combined primary extract fraction and secondary extract fraction, assuming the total equals 100%), was 37.65%. For TFC, the relative contribution in the secondary fraction was 34.38%. For glycerol–water extraction (65%), the relative contributions of TPC and TFC were 42.43% and 43.18%, respectively. When nanowater was used as the extractant, the relative contributions of TPC and TFC in the secondary peppermint extract were 38.09% and 30.41%, respectively. For 50% ethanol–water extraction, the relative contributions of TPC and TFC in secondary extracts were 41.48% and 40.20%. In the case of oil-based extraction, the relative contributions of TPC and TFC were 48.34% and 42.00%, respectively.

The presentation of relative contributions of active compounds in primary and secondary extracts constitutes a semi-quantitative approach; nevertheless, it clearly indicates that secondary extraction makes a substantial contribution to the overall number of polyphenols and flavonoids recovered from the investigated herbs across different extraction systems. In many cases, the relative share of the analyzed active compounds in the secondary fraction exceeded 30–40%, highlighting the importance of performing secondary extraction, which may provide valuable constituents suitable for various applications. Overall, the above data demonstrate that post-extraction residues represent a valuable source of biologically active compounds.

### 2.4. Extraction Yield

**Figure 10:**
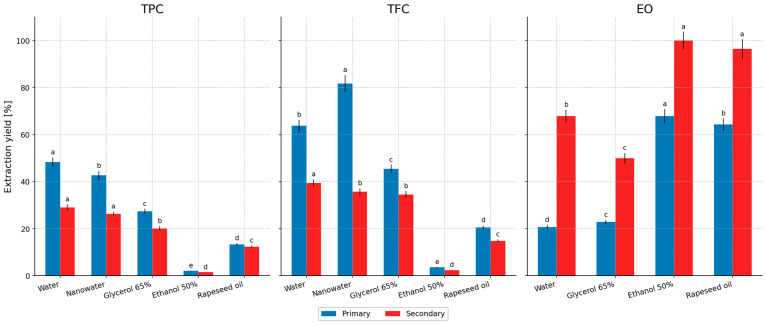
Extraction yield [%] for TPC, TFC, and EO in processes conducted using the following solvents: water, glycerol–water, nanowater, rapeseed oil, and ethanol–water, under the conditions 10 min and 100 °C (and 50 °C for the 50% ethanol–water system). Error bars represent standard deviation (SD) from three independent replicates. Different letters (a, b, c, etc.) indicate statistically significant differences at *p* < 0.05.

Figure 10 presents the extraction yields (for TPC, TFC, and EO) obtained for all tested solvents (water, glycerol–water, nanowater, rapeseed oil, and ethanol–water) under the most favorable process conditions: 10 min and 100 °C (except for the 50% ethanol–water system, for which 50 °C was applied). A 70% methanolic extract was used as the reference extract, for which 100% extraction efficiency was assumed with respect to TPC and TFC [78]. Importantly, this reference was obtained by exhaustive continuous solvent extraction in a Soxhlet apparatus (12 h); therefore, the yields reported for the 10 min infusion-type protocols express the percentage recovery achieved relative to an exhaustive benchmark, rather than the absolute extraction strength of the tested solvents.

For TPC, the highest yields (for both primary and secondary extraction) were obtained for water: 48.31% and 29.11%, respectively. The next most effective solvent was nanowater: 42.69% and 26.27%. The lowest values were recorded for the ethanol–water system (50%): 2.14% and 1.51%. For glycerol–water extraction (65%), yields of 27.44% and 20.22% were obtained, whereas for rapeseed oil the values were 13.26% and 26.27%.

For TFC, water and nanowater were also the best extractants. In the case of water, yields of 63.75% and 39.47% were obtained, while for nanowater the corresponding yields were 81.73% and 14.88%. Again, the ethanol–water system (50%) showed the lowest yields: 3.55% and 2.38%. It is worth emphasizing the relatively high yields achieved using glycerol–water (65%): 45.47% and 34.56%, whereas oil-based extraction resulted in 20.56% and 14.88%.

Notably, ‘EO yield’ in this study represents a depletion-based recovery calculated from distillation of plant material before and after extraction (Equation (2)), rather than direct quantification of essential oil concentration in each liquid extract. With respect to EO, the highest yields were obtained for 50% ethanol and rapeseed oil. For oil extraction, yields were 64.29% (primary) and 96.43% (secondary), while for 50% ethanol extraction the values were 67.86% and 100%, respectively. For the glycerol–water system (65%), EO yields were 22.86% and 50.00%, while for aqueous extraction they were 20.71% and 67.86%.

Overall, these yield profiles indicate a clear solvent-dependent selectivity between polar (TPC/TFC) and volatile/lipophilic (EO) fractions. Notably, the higher secondary yield observed for rapeseed oil in the case of TPC, as well as the pronounced drop in secondary TFC yield for nanowater, suggest that mass-transfer constraints and matrix–solvent interactions differ substantially across solvent systems. These effects are discussed below in the context of solvent polarity, viscosity-driven diffusion limitations, and the kinetics of short, infusion-type extraction.

Building on the solvent-dependent yield patterns shown in Figure 10, the observed selectivity can be mechanistically attributed to differences in solvent polarity and hydrogen-bonding capacity (governing the solubilization of phenolic acids and flavonoids), as well as to viscosity-controlled mass transfer and phase affinity for volatile terpenoids (governing EO recovery) [4,7,32]. In short, water and glycerol–water systems preferentially promote the release of polar constituents (TPC/TFC) from the leaf matrix during short infusion-like extraction, whereas ethanol–water and edible oil provide a markedly higher driving force for the partitioning of essential-oil constituents into the liquid phase [79,80,81]. Importantly, because the yields are expressed relative to the 70% methanolic reference extract (assumed as 100% for TPC and TFC), the reported percentages represent recovery under mild, low-processed conditions and do not constitute an absolute ranking of solvent extraction strength.

The extraction yields shown in Figure 10 clearly demonstrate a process-driven trade-off typical of low-processed extraction systems: aqueous and glycerol–water media favor the recovery of polar phytochemicals (TPC and TFC), whereas ethanol–water (50%) and rapeseed oil preferentially recover the volatile/lipophilic fraction (EO). This pattern is consistent with basic solubility and mass-transfer principles and should be considered in relation to the adopted definition of extraction yield. For the purposes of the present work, a 70% methanolic Soxhlet extract (12 h) was treated as an exhaustive reference and set to 100% yield for TPC and TFC; therefore, the yields obtained in 10 min infusion-type extractions represent relative recovery under mild conditions rather than an absolute measure of solvent “strength”.

For TPC, the highest yields were obtained for water (48.31% primary; 29.11% secondary), followed by nanowater (42.69%; 26.27%). This agrees with the predominantly polar nature of peppermint phenolics, which are efficiently released into aqueous media at elevated temperatures under infusion-like conditions [77,82]. Importantly, short infusion-type extraction typically exhibits rapid early release followed by a kinetic plateau governed by intraparticle diffusion and partition equilibrium; therefore, a second extraction step with fresh solvent can recover an additional fraction more efficiently than prolonging the same extraction time [4,7]. This mechanistic rationale supports the selection of 10 min as an operational optimum for the primary step and explains the meaningful recoveries still observed in the secondary extraction.

The glycerol–water system (65%) provided intermediate yields for TPC (27.44%; 20.22%) but relatively high yields for TFC (45.47%; 34.56%). Glycerol-based mixtures are increasingly recognized as food-grade green solvents capable of solubilizing phenolic antioxidants while improving extract stability; however, their elevated viscosity can limit mass transfer during short contact times, particularly in infusion-type protocols, unless temperature is sufficiently high to reduce viscosity and enhance diffusivity [4,7,32]. Literature on heated glycerol–water extraction repeatedly highlights these viscosity/temperature effects and supports glycerol’s applicability within green chemistry frameworks, especially when process conditions are optimized to mitigate transport limitations [7,83].

The very low TPC/TFC yields observed for 50% ethanol–water (2.14%/1.51% for TPC and 3.55%/2.38% for TFC) are consistent with the applied process temperature (50 °C rather than 100 °C), which reduces swelling of the plant matrix and slows diffusion/desorption of polar phenolics compared with hot-water extraction [4,82]. From a process-engineering standpoint, solvent choice is inherently a compromise among polarity/solvation capacity, viscosity and mass-transfer coefficients, consumer safety, and technologically acceptable temperatures; hence, within the present design, the ethanol system appears better positioned as a “green” solvent targeting the volatile/lipophilic fraction rather than rapid phenolic recovery under infusion conditions [32,33,79]. This interpretation is also consistent with published studies on peppermint hydroalcoholic or alcoholic extracts, although direct numerical comparison should be made with caution because most literature protocols involve much longer extraction times, different solvent-to-solid ratios, and final results expressed per dry mass of plant material rather than as relative recovery against an exhaustive reference. Farnad et al. [84] reported that alcoholic peppermint extracts were rich in phenolic constituents and exhibited substantial antioxidant activity, confirming that alcohol-based systems can be effective when used in extraction-oriented rather than infusion-type protocols. Likewise, Fatemi et al. [85] reported that a hydroalcoholic peppermint extract retained measurable flavonoid content and antioxidant activity under their experimental conditions, confirming that hydroalcoholic systems can preserve bioactive properties of peppermint extracts. However, because their study addressed a different processing context, it should not be directly compared numerically with the present short infusion-type extraction model. Therefore, the very low relative TPC/TFC yields obtained in the present study for 50% ethanol are more reasonably attributed to the short, low-processed extraction format than to a generally poor suitability of hydroalcoholic solvents for peppermint.

This interpretation is strongly supported by the EO yields: the highest recoveries were achieved with 50% ethanol (67.86% primary; 100% secondary) and rapeseed oil (64.29%; 96.43%). Such performance is expected because peppermint essential-oil constituents (mainly monoterpenes and oxygenated monoterpenes) exhibit much higher affinity for alcohol and lipid phases than for water [79,80,81]. Moreover, the two-step design effectively depleted the volatile fraction in the ethanol system (100% after secondary extraction), emphasizing a practical application route: ethanol–water and edible oils can serve as safe “end-use” solvents for functional extracts enriched in aroma/volatile constituents. The broader concept of edible oils as non-toxic solvents for green extraction of lipophilic/aromatic compounds is also well documented [79,80].

A key novelty highlighted by these results is that a substantial proportion of bioactives remains in the post-extraction biomass and can be recovered by secondary extraction. In Figure 10, secondary extraction still yields meaningful percentages for TPC and TFC, and elsewhere in the manuscript the relative contribution of secondary fractions frequently exceeds 30–40% across solvent systems. This directly supports circular-economy valorization: even simple, low-energy secondary extraction can convert a “waste” residue into a valuable feedstock for food, nutraceutical, or cosmetic applications, aligning with current green extraction and biomass valorization strategies [32,33,86].

Overall, the presented yields suggest that a sequential strategy (e.g., water or glycerol–water for TPC/TFC, followed by ethanol–water or edible oil for EO) may be technologically more rational than attempting to maximize all fractions with a single solvent, while maintaining food-grade solvent compliance and enabling application-oriented tailoring of extract profiles [32,79].

### 2.5. Evaluation of Antioxidant Activity of the Extracts

**Figure 11:**
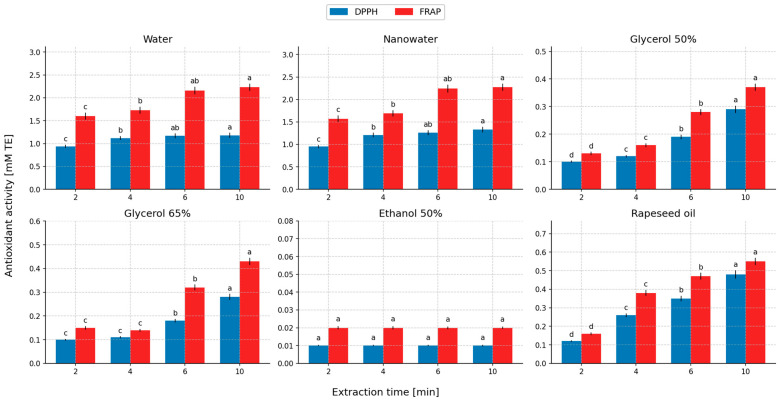
Antioxidant activity as a function of extraction time. Error bars represent standard deviation (SD) from three independent replicates. Different letters (a, b, c, etc.) indicate statistically significant differences at *p* < 0.05.

**Figure 12:**
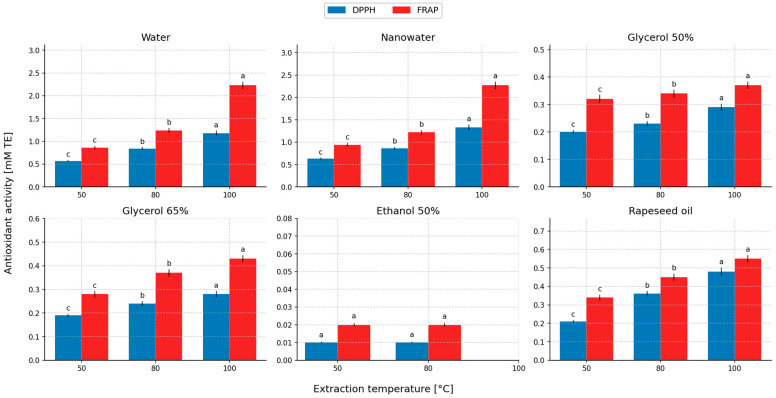
Antioxidant activity as a function the initial temperature of the extractant used in the extraction process. Error bars represent standard deviation (SD) from three independent replicates. Different letters (a, b, c) indicate statistically significant differences at *p* < 0.05.

Figure 11 and Figure 12 present the antioxidant properties of the extracts as a function of extraction time and the initial temperature of the extractant used in the extraction process. In this section, the comparison of antioxidant activity between extracts is treated as a functional comparison of the obtained liquid systems and is interpreted together with TPC/TFC recovery, rather than as an absolute intrinsic property of the solvent itself.

The antioxidant activity determined by the DPPH and FRAP assays decreased in the following order of extracts (DPPH/FRAP, respectively): aqueous systems (nanowater and water): nanowater (1.33/2.27 mM TE) > water (1.18/2.23 mM TE) > rapeseed oil (0.48/0.55 mM TE) > 50% glycerol (0.29/0.37 mM TE) ≈ 65% glycerol (0.28/0.43 mM TE) > 50% ethanol (0.01/0.02 mM TE). Extracts obtained using water (nanowater) as the extractant exhibited high antioxidant activity, whereas ethanol–water extracts showed the lowest values compared with the other solvents, which was consistent with the concentrations of polyphenols and flavonoids in these extracts. The antioxidant effects of peppermint are attributed mainly to phenolic compounds and, to some extent, to essential oil constituents [87,88]. The obtained results of antioxidant activity are qualitatively consistent with literature data, according to which aqueous and water-hydrophilic extracts from peppermint show the highest activity against the DPPH radical and in tests based on reducing capacity, which is connected with the high content of phenolic compounds. In the present study, the aqueous systems (nanowater and water) achieved the highest DPPH/FRAP values, whereas the 50% ethanol system showed minimal activity, which corresponded to very low levels of TPC and TFC in these extracts. This relationship remains consistent with the general picture presented in publications concerning peppermint, in which the antioxidant activity of extracts was positively associated with the share of phenolics soluble in more polar extraction systems [34,35,36,37,87,88].

### 2.6. EO Content in Post-Extraction Material

Peppermint leaves were found to contain 1.40% *v*/*w* EO (Table 2). Duband et al. [89] reported a higher EO content than that obtained in the present study (2.40% *v*/*w* EO in fresh peppermint leaves). Adaszyńska et al. [90] reported an EO level of 2.10% *v*/*w*. In the study by Freire et al. [91] EO content in peppermint leaves ranged from 1.00 to 2.00%. Kowalski et al. [92] reported an EO content of 2.05% *v*/*w*. The EO content in plant materials depends on multiple factors, including environmental conditions (temperature, light intensity, air humidity), agrotechnical factors (soil and fertilization, harvest date), and genetic factors [93]. According to Pharmacopea Helvetica VII [94] the EO content in peppermint leaves should not be lower than 1.00% *v*/*w*. The Polish Pharmacopoeia VIII [95] states that peppermint leaves should contain no less than 1.50% *v*/*w* of essential oil, and that the minimum level should exceed 1.20% *v*/*w*. These data indicate that the EO content obtained from peppermint leaves in the present study was appropriate. Moreover, Kowalski et al. [96] showed that ultrasound-assisted pre-maceration increased the EO yield from peppermint leaves from 1.32% *v*/*w* to 1.46% *v*/*w*. The EO content reported in the present study falls within the indicated standards. It is worth emphasizing that although the absolute EO content in the raw material examined in this study was lower than some of the values reported for fresh or differently obtained plant materials [89,90,91,92], it still remained within the pharmacopoeial limits and was comparable to the range reported for peppermint leaves in other studies. This means that the analyzed raw material may be regarded as representative for the evaluation of changes in essential oil content and recovery during primary and secondary extraction. Thus, the observed differences between the solvents should be interpreted mainly as an effect of the applied extraction conditions, and not as a result of unusually low quality of the starting material.

Herbal extracts contain volatile constituents that confer the characteristic aroma of herbal infusions and extracts. The presence of volatile substances contributes to the health-promoting value of such preparations; therefore, the content of this group of constituents is important. Due to the low solubility of EO in water, its concentration in aqueous extracts is generally low; however, the high biological activity of these volatile compounds may exert beneficial effects even at low concentrations.

Given that the extraction systems applied in this study differed in their affinity for EO, a research hypothesis was adopted that the post-extraction plant material (post-extraction waste) contains significant amounts of bioactive compounds, including EO, which could be recovered for various purposes. To verify this hypothesis, EO content in post-extraction plant materials was determined, as shown in Table 2.

The percentage content of EO in the post-extraction peppermint leaf materials varied and depended on the solvent used in the previous extraction step (Table 2). Differences were also observed between EO isolated from the primary and post-extraction materials. The highest EO content after primary extraction was observed when water (1.35–1.11% *v*/*w*) and 65% glycerol–water solution (1.15–1.03% *v*/*w*) were used, depending on the applied time–temperature parameters. The lowest EO contents were recorded after oil extraction (0.50–0.73% *v*/*w*) and 50% ethanol extraction (0.45–0.55% *v*/*w*), indicating the high extraction efficiency of these solvents toward essential oil (Figure 10). After secondary extraction using rapeseed oil, EO content ranged from 0.01 to 0.05% *v*/*w*. In contrast, secondary extraction with the ethanol–water system enabled complete extraction of the EO fraction, resulting in the absence of these constituents in the post-extraction material. The highest EO contents after secondary extraction, similarly to the primary extraction, were obtained for water (0.45–0.55% *v*/*w*). The results indicate that after primary extraction, EO contents for aqueous and glycerol–water (65%) systems were comparable. EO content in plant materials after primary extraction was higher when water was applied compared with glycerol–water extraction, suggesting better extraction properties of glycerol for this plant matrix.

Table 3 presents the remaining EO in plant material after primary and secondary extraction, expressed as relative percentage contributions assuming that the initial EO content equals 100%. The highest levels of EO remaining in the plant material were observed after primary and secondary water and glycerol–water extractions. After primary water extraction, the remaining EO ranged from 79.29 to 96.43%, depending on time and temperature. For primary glycerol–water extraction, the remaining EO ranged from 73.57 to 82.14%. The lowest remaining EO levels after primary extraction were observed for oil extraction (35.71–52.14%) and 50% ethanol–water extraction (32.14–39.29%). After secondary extraction, the plant material treated with the 50% ethanol–water system contained no essential oil, indicating complete extraction of the volatile fraction. Only small amounts of EO remained after secondary oil extraction, corresponding to 0.71–3.57% in relative terms. Similarly to the primary extraction, the highest EO contents after secondary extraction were observed for water extraction, reaching 32.14–39.28% in relative terms.

Table 4 presents EO levels (expressed as relative percentage contribution with respect to total EO content) released during extraction from plant materials after primary and secondary extraction. The highest EO transfer into extracts was obtained for oil extraction (47.86–64.29%) and ethanol–water extraction (60.71–67.86%). The lowest EO amounts in the extracts were observed for water extraction (3.57–20.71%) and glycerol–water extraction (17.86–26.43%). After secondary extraction using the 50% ethanol–water system, the entire volatile oil fraction was extracted (100%). Similarly high EO recovery was observed for the oil extraction variant, with relative contributions of 96.43–99.29% compared to the initial EO content. The lowest EO release after secondary extraction was recorded for water and glycerol–water variants. For water extraction, relative contributions ranged from 60.72 to 67.86%, whereas for glycerol–water extraction they ranged from 39.29 to 57.14%.

In summary, ethanol–water and oil-based extractions showed the highest efficiencies, and in these cases two extraction steps enabled recovery of virtually the entire volatile essential oil fraction. In contrast, the remaining solvents exhibited lower yields, which is related to the physicochemical properties of EO: essential oils are nearly insoluble in water and only slightly soluble in glycerol, while they dissolve readily in ethyl alcohol and fats [95]. In those studies, the contents of selected active compounds in primary and secondary herbal extracts were evaluated, and substantial amounts of bioactive compounds were detected in secondary infusions. Furthermore, the authors observed that post-extraction herbal material contained significant amounts of EO relative to the initial content. It should be emphasized that the approach presented in the current study is novel and may be developed further in future research.

### 2.7. Characterization of the EO Composition in the Initial Plant Material and Post-Extraction Residues

Table 5 presents the percentage contribution of individual components of the essential oil (EO) isolated from the initial plant material—peppermint leaves. Qualitative analysis revealed the presence of 77 compounds, predominantly belonging to the terpene group and aromatic hydrocarbons.

Chromatographic analysis of EO isolated from peppermint enabled the identification of 77 compounds, accounting for 99.80% of the oil (Table 5). Among the constituents present in peppermint oil, aromatic hydrocarbons (46.75%) and aliphatic alcohols (37.66%) predominated, while ketones were also detected (7.79%). The main components of peppermint EO were menthol (36.10%), menthone (32.06%), isomenthone (6.32%), neomenthol (2.19%), pulegone (1.72%), piperitone (1.46%), and menthyl acetate (10.32%). Similar results were reported by Mahboubi and Kazempour [99], who identified 33 EO components in peppermint oil accounting for 99.8% of the total oil, with menthol (36.9%), menthone (28.8%), and methyl acetate (4.5%) as the dominant constituents, followed by carvone (3.8%), neomenthol (3.8%), 1,8-cineole (3.8%), and limonene (3.29%). Kazem et al. [100], demonstrated that the most important EO constituents of peppermint were menthol (39.81%), menthone (19.55%), neomenthol (8.83%), menthyl acetate (8.64%), and 1,8-cineole (5.81%).

Higher concentrations of individual constituents were reported by Saharkhiz et al. [101], who identified 17 EO components representing 99.37% of peppermint oil, with menthol (53.28%), menthyl acetate (15.10%), 1,8-cineole (6.69%), and menthone (2.45%) as dominant constituents.

In many studies, menthol has been reported as the main component of peppermint oil [102,103,104], followed by menthone and limonene [102,104]. In one study, β-terpinene and piperitone oxide were identified as dominant components [105]. The chemical composition of peppermint EO obtained in the present study is comparable to the results reported by Iscan et al. [102] with menthol and menthone as the predominant constituents.

Table 6 presents the composition characteristics of peppermint EO isolated from post-extraction materials (after primary and secondary extraction). Chromatographic analysis revealed both quantitative and qualitative differences between EOs obtained from post-extraction materials and EO isolated from the initial plant material (Table 6).

In peppermint EO isolated from plant material after prior primary aqueous extraction (10 min, 100 °C), 57 compounds were identified (Table 7), accounting for 95.3% of the oil. These included menthol (31.46%), menthone (25.89%), isomenthone (5.92%), neomenthol (2.81%), pulegone (0.24%), piperitone (1.24%), and menthyl acetate (10.12%).

For EO obtained after glycerol–water extraction (65%) under identical conditions (100 °C, 10 min), 68 compounds were identified, representing 91.12% of the oil, with menthol (24.45%), menthone (22.81%), isomenthone (6.01%), neomenthol (2.48%), pulegone (0.26%), piperitone (1.12%), and menthyl acetate (12.56%) as dominant constituents.

After extraction using rapeseed oil under the same conditions, 64 compounds were identified (88.7% of the oil), including menthol (28.39%), menthone (25.46%), isomenthone (7.10%), neomenthol (2.77%), pulegone (0.31%), piperitone (1.51%), and menthyl acetate (13.77%).

Following ethanol–water extraction (50%, 10 min, 50 °C), 75 EO compounds were identified, representing 98.7% of the oil, with menthol (20.69%), menthone (30.25%), isomenthone (6.14%), neomenthol (1.56%), pulegone (0.19%), piperitone (1.47%), and menthyl acetate (5.98%).

Qualitative analysis of EO was also performed for plant materials after secondary extraction (Table 7). After double aqueous extraction (10 min, 100 °C), 56 compounds were identified (94.2% of the oil), including menthol (23.46%), menthone (19.80%), isomenthone (4.52%), neomenthol (1.56%), pulegone (0.16%), piperitone (0.97%), and menthyl acetate (3.17%).

Double glycerol–water extraction (65%) under the same conditions yielded 64 compounds (90.12% of the oil), with menthol (7.45%), menthone (11.85%), isomenthone (3.12%), neomenthol (0.85%), pulegone (1.46%), piperitone (1.52%), and menthyl acetate (3.12%).

After double oil extraction (100 °C, 10 min), 64 compounds were identified (87.6% of the oil), including menthol (15.68%), menthone (10.56%), isomenthone (2.56%), neomenthol (1.03%), pulegone (0.05%), piperitone (0.89%), and menthyl acetate (2.12%).

Following double ethanol–water extraction (50%, 10 min, 50 °C), 71 compounds were identified, representing 95.12% of the oil, with menthol (6.14%), menthone (18.50%), isomenthone (5.17%), neomenthol (1.05%), pulegone (0.05%), piperitone (0.41%), and menthyl acetate (4.12%).

In summary, the composition of “recovered” EOs from post-extraction plant materials depended directly on the chemical nature of the solvent previously applied. Because EOs are readily soluble in alcohols and fats, these solvents efficiently isolate volatile constituents and markedly modify the composition of residual EO fractions in plant tissues. Temperature and extraction time were also key factors affecting EO extraction and the composition of the remaining oil fraction.

Previous studies rarely address the detailed characterization of EOs recovered from post-extraction materials as a function of solvent system, highlighting the novelty of this dissertation. Wyrostek et al. [47] reported compositional changes in EOs recovered after brewing and proposed potential applications of such oils.

Comparable observations were reported for clove buds, where 33–57% of EO was recovered after primary extraction and 26–42% after secondary extraction, depending on the solvent used [106] with significant differences in eugenol and E-caryophyllene content relative to the original oil.

**Figure 13:**
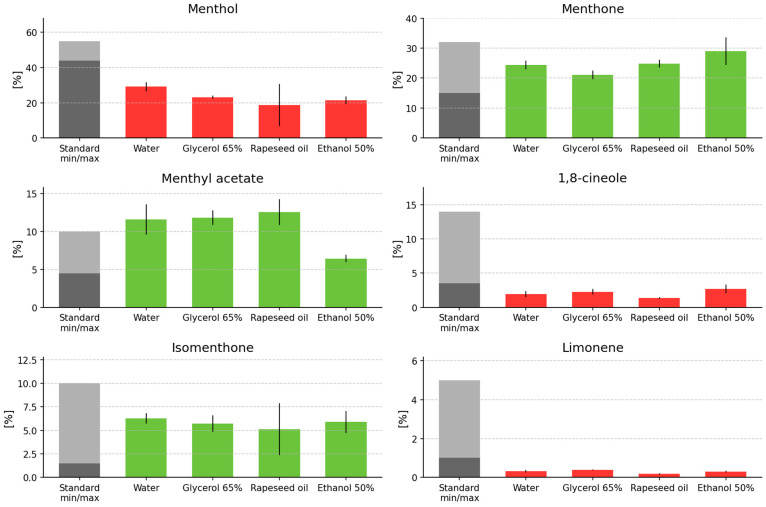
Profiles of the main EO constituents of peppermint for the investigated solvents and the corresponding normative requirements according to the European Pharmacopoeia [107]. The dark grey segment represents the lower limit of the normative range (Min), whereas the combined height of the dark and light grey segments corresponds to the upper limit of the normative range (Max). Colored bars represent the mean percentage contents of the indicated constituents in EO isolated from post-extraction plant material obtained with the investigated solvents; green bars indicate values within or above the normative range, whereas red bars indicate values below the normative lower limit. Error bars represent standard deviation (SD).

Quality parameters of EOs, which have particular applications across various industrial sectors, are defined by relevant normative standards (pharmacopeial and ISO standards), and compliance with these requirements is indicative of good commercial quality. Therefore, to assess the quality of the oils obtained from the post-extraction plant materials investigated in this study, a qualitative and quantitative verification was performed against the compositional limits specified for peppermint essential oil in the European Pharmacopoeia [107], including the required ranges for selected major constituents (Figure 13). It should also be emphasized that, in the qualitative assessment of oils obtained from post-extraction materials, the primary determinant of composition is the quality of the initial plant material, which may not always meet the requirements of the relevant standards; consequently, there is a high probability that EO obtained via secondary distillation will also fail to comply with the standards. However, in the present work, a direct characterization of the investigated plant raw materials in terms of existing normative requirements was not undertaken; instead, the evaluation was limited solely to the “recovered” essential oil in this respect. In the plots illustrating compliance with the relevant requirements (Figure 13), deviations from normative specifications for the “recovered” essential oils are marked using red-colored bars.

In peppermint oil recovered from post-extraction materials, the contents of individual constituents varied depending on the solvents applied and the time–temperature parameters, as well as on the number of extraction steps performed (primary vs. secondary). For example, in the investigated peppermint oils, menthone content ranged from 19.83% to 36.10%, whereas menthol content ranged from 1.71% to 31.46%. In some cases, an increasing trend for a given constituent was observed compared with its content in the initial material; for instance, the isomenthol content in the initial material was 0.33%, while in the oil isolated after glycerol–water extraction (65%) the contribution of this constituent was 0.48–0.57%, depending on the applied time and temperature conditions. An opposite, decreasing trend relative to the initial material was observed for piperitone: its content in EO from the initial material was 1.46%, whereas in the oil obtained from material after glycerol–water extraction (65%) the contribution of this constituent ranged from 0.85 to 1.12%. Considering the levels of identified constituents in peppermint EO isolated after primary and secondary extraction, substantial decreases in the contribution of these constituents were observed in oils obtained after secondary extraction.

The qualitative evaluation of peppermint EO according to the requirements of the European Pharmacopoeia [107] is based on the analysis of relative amounts of: menthol—44–55% (free alcohols, expressed as menthol); ketones (expressed as menthone)—15–32%; esters (expressed as menthyl acetate)—4.5–10%; cineole—3.5–14%; isomenthone—1.5–10%; menthofuran—1–9%; limonene—1–5%; not more than 4% pulegone or 1% carvone; and viridiflorol at approximately 0.5%. Figure 12 presents the profiles of the main EO constituents isolated from peppermint post-extraction material for the solvents applied in this study.

It was observed that for constituents such as menthol, limonene, and 1,8-cineole, their contents in EO isolated from post-extraction material did not meet the standard requirements for all solvent variants. The highest menthol level was obtained when water was used as the solvent, reaching 29.10%, i.e., 14.90% below the lower threshold value (44%) specified in the standard. The lowest menthol contribution was recorded in EO from post-extraction material when rapeseed oil was used as the extractant (18.70%), i.e., 25.30% below the lower threshold value. For glycerol (65%) used as the solvent, menthol amounted to 23.18% (20.82% below the lower threshold value), whereas for ethanol (50%) used as the extractant, the contribution was 21.43% (22.57% below the lower threshold value).

For limonene, with a lower threshold value of 1%, the contribution of this constituent was: 0.38% (glycerol) > 0.32% (water) > 0.29% (ethanol 50%) > 0.17% (oil). For 1,8-cineole, with a threshold value of 3.5%, the contributions were: 2.71% (ethanol 50%) > 2.28% (glycerol) > 1.96% (water) > 1.36% (oil). In contrast, menthone content in the analyzed oils was within the normative range (15–32%), namely: 29.00% (ethanol 50%) > 24.80% (oil) > 24.39% (water) > 21.05% (glycerol). Isomenthone content also remained within the normative range (1.5–10.0%), with the following percentage contributions depending on the solvent used in the preceding extraction step: 6.29% (water) > 5.90% (ethanol 50%) > 5.72% (glycerol) > 5.12% (oil). For menthyl acetate, percentage contributions higher than those specified in the standard (4.50–10.00%) were observed in oils isolated using the investigated solvents, except for ethanol (50%): 12.56% (oil) > 11.82% (glycerol) > 11.60% (water) > 6.44% (ethanol 50%).

## 3. Materials and Methods

### 3.1. Plant Material

Peppermint leaves (*Mentha* × *piperita* L.) were used in the study and were obtained from a single production batch from the company Dary Natury Sp. z o.o. (Koryciny 73, 17-315 Grodzisk, Poland). The raw material was appropriately dried and properly packaged. The plant material was standardized by pooling and grinding with the use of a laboratory analytical mill type A11 Basic (IKA-Werke GmbH, Staufen, Germany) to obtain a homogeneous fraction (5.6 mm sieve). The moisture content of the prepared material was determined using a WPS 50 SX moisture analyser (RADWAG, Radom, Poland) and averaged 9.2%, confirming that the raw material was properly dried and sufficiently stable for reproducible extraction experiments. Subsequently, the prepared plant material was stored in plastic packaging under conditions limiting exposure to light and moisture.

### 3.2. Chemicals

All chemical reagents used in the study were of analytical grade (a.g.) or higher, unless otherwise stated.

The following extraction solvents were used: distilled water, vegetable glycerol (≥99.5%, Ph. Eur., Galvet, Łambinowice, Poland), ethanol and methanol (a.g., POCH S.A., Gliwice, Poland), rapeseed oil (Kujawski, Kruszwica, Poland), as well as plasma-treatednanowater—low-temperature plasma-treated tap water; manufacturer-declared ROS enrichment (including singlet oxygen) (Nantes Medical, Bolesławiec, Poland).

For the determination of total polyphenol content (TPC) and total flavonoid content (TFC), the following reagents were used: Folin–Ciocalteu reagent, sodium carbonate, sodium nitrate(V), aluminum chloride, and sodium hydroxide (all a.g., POCH S.A., Gliwice, Poland). Gallic acid (≥98%) and epicatechin (≥98%) were used as calibration standards (Sigma-Aldrich, Buchs, Switzerland).

Antioxidant activity was determined using the DPPH and FRAP methods. For the DPPH assay, 1,1-diphenyl-2-picrylhydrazyl (DPPH, ≥97%) and the reference standard Trolox (6-hydroxy-2,5,7,8-tetramethylchroman-2-carboxylic acid, ≥98%) were used (Sigma-Aldrich, Buchs, Switzerland). For the FRAP assay, 2,4,6-tripyridyl-s-triazine (TPTZ, ≥98%), iron(III) chloride hexahydrate (FeCl_3_·6H_2_O, ≥99%), and acetate buffer components were applied (Sigma-Aldrich, Buchs, Switzerland).

For essential oil analyses and preparation of samples for chromatographic analyses, acetone, xylene, and hexane (a.g., POCH S.A., Gliwice, Poland), anhydrous sodium sulfate (a.g., Chempur, Piekary Śląskie, Poland), as well as n-alkane retention index standards: dodecane and nonadecane (≥99%, Sigma-Aldrich, Buchs, Switzerland) were used.

All aqueous solutions were prepared immediately before use using distilled water.

### 3.3. Extraction

A classical extraction procedure corresponding to the process of preparing tea infusions according to the PN-ISO 3103 standard [3] was applied, with modifications in terms of solvent type and temperature. For this purpose, 2 g of plant material were weighed into a 150 mL beaker and poured with 100 mL of an appropriate solvent. In the case of water, 65% and 50% aqueous glycerol solutions, oil, and nanowater, initial extraction temperatures of 50 °C, 80 °C, and 100 °C were used, whereas for 50% ethanol, a temperature of 50 °C and room temperature were applied. Subsequently, the individual systems were subjected to gentle mixing at 130 rpm (IST-3075R shaker, Jeio Tech, Daejeon, Republic of Korea). Thus, the extraction experiments were performed at a plant material-to-solvent ratio of 1:50 (*w*/*v*) in 150 mL glass beakers under gentle agitation, and all extraction variants were carried out in three independent replicates. For the determination of essential oil content in the extracts, the mass of the raw material and the volume of the solvent were increased tenfold.

**Figure 14:**
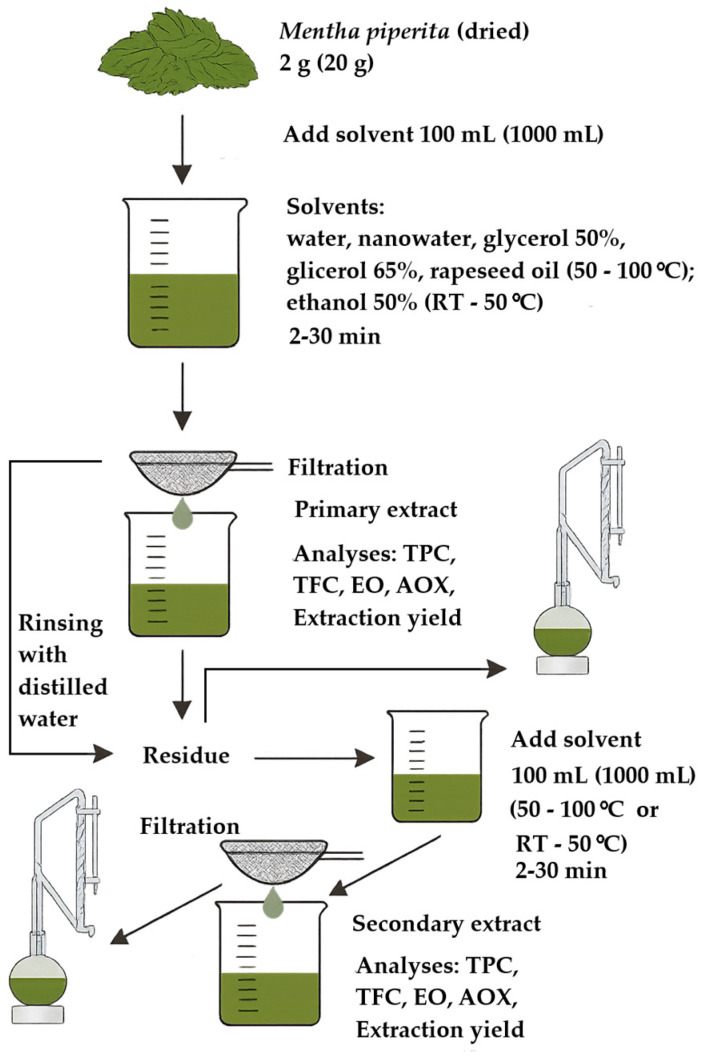
Schematic diagram illustrating the preparation of the extracts, which were subsequently evaluated according to the described protocol.

The extraction process was carried out under different time variants ranging from 2 to 30 min. Next, the obtained (primary) extracts were separated from the post-extraction plant material and used for the determination of polyphenol content, flavonoid content, essential oil content, and antioxidant capacity. In addition, the post-extraction plant material was rinsed with distilled water at room temperature and subjected again to the extraction process using various extractants under analogous temperature and time conditions as described above (secondary extraction). The obtained secondary extracts were evaluated in the same manner as the primary extracts, with the determination of extraction yield. Table 8 presents a summary of the solvents used along with the extraction time and temperature. Figure 14 shows a schematic diagram illustrating the preparation of the extracts.

### 3.4. Methanolic Extraction

Methanolic extraction (70%) was treated as an exhaustive extraction, which was carried out until practically the entire group of the analyzed biologically active compounds had been extracted [78]. Extraction using 70% methanol as the extractant was performed by continuous solvent extraction in a Soxhlet apparatus. In order to obtain the extract, 2 g of the investigated plant raw materials and 100 mL of 70% methanol were used. The extraction was carried out for 12 h, after which the obtained extract was filtered and stored at a temperature of approximately 4 °C. All extracts were adjusted to an equal final volume of 100 mL. The duration of methanolic extraction was set at 12 h, because after this time the contents of the compounds in the analyzed extracts did not show statistically significant differences. Moreover, the post-extraction material was subjected to secondary extraction to verify the presence of active substances, by assessing the content of the analyzed components in the plant material and confirming the absence of polyphenol and flavonoid fractions.

### 3.5. Determination of Total Phenolic Compound Content

The total phenolic content (TPC) of the analyzed extracts was quantified using a spectrophotometric assay performed at 765 nm, based on the method originally described by Singleton and Rossi, with minor modifications [46,108]. The applied modification consisted of proportional changes in the volumes of the reagents used in the reaction mixture.

Briefly, 0.02 mL of the tested extract was transferred into a 5 mL volumetric flask and diluted with 3.16 mL of distilled water. Subsequently, 0.10 mL of Folin–Ciocalteu reagent was added, the solution was thoroughly mixed, and left to react for 5 min. After this period, 0.60 mL of saturated sodium carbonate (Na_2_CO_3_) solution was introduced, followed by mixing and incubation at 40 °C for 30 min.

The absorbance was recorded against a reagent blank prepared analogously, except that 0.02 mL of distilled water was used instead of the extract. TPC values were expressed as gallic acid equivalents (GAE), using gallic acid as the reference standard (Sigma-Aldrich, St. Louis, MO, USA; ACS reagent, ≥98.00%). Quantification was performed using a calibration curve constructed within the concentration range of 10–60 mg/L (10, 20, 30, 40, 50, and 60 mg/L). Prior to analysis, each sample was appropriately diluted to ensure that the absorbance readings fell within the linear range of the calibration curve. All measurements were carried out in triplicate. The results were expressed as mg GAE/L of extract, since the study focused on low-processed liquid extracts and not on dry-matter-normalized plant material.

### 3.6. Determination of Total Flavonoid Content

The total flavonoid content (TFC) of the analyzed extracts was assessed by a spectrophotometric method at 510 nm, using a protocol adapted from Karadeniz et al., with slight modifications [46,109].

For the assay, 1.0 mL of the tested extract was placed in a 10 mL volumetric flask. Next, 5.0 mL of redistilled water was added, followed by 0.3 mL of a 5% (*w*/*w*) aqueous sodium nitrate solution. The mixture was allowed to react for 5 min. Subsequently, 0.6 mL of a 10% (*w*/*w*) aqueous aluminum chloride hexahydrate solution was introduced and the contents were mixed thoroughly. After an additional 5 min, 2.0 mL of 1 M aqueous NaOH solution was added, and the flask was filled up to the mark with redistilled water.

The absorbance of the resulting solutions was measured at 510 nm against a blank. The results were expressed as epicatechin equivalents (ECE), using epicatechin as the reference compound (Sigma-Aldrich; ACS reagent, ≥98.00%). Quantification was performed based on a calibration curve prepared with epicatechin standards within the range of 10–400 mg/L. Prior to measurement, the samples were diluted as necessary to ensure that the readings fell within the linear range of the calibration curve. All determinations were performed in triplicate. The results were expressed as mg ECE/L of extract, in accordance with the concentration-based evaluation of the obtained liquid extracts.

### 3.7. Determination of Essential Oil Content in Plant Material

The essential oil content (EO) in the plant material was determined by steam distillation, in accordance with the pharmacopoeial procedure [95]. Approximately 20 g of plant raw material (with an accuracy of 0.1 g), both before and after extraction, was weighed into a 1000 mL flask and mixed with 400 mL of water. The prepared flask was connected to the distillation apparatus, the receiver was filled with water, and cooling was started. Distillation was carried out for 3 h, calculated from the moment the first drop of distillate was collected.

The obtained essential oil was transferred to the microscale section of the apparatus, and the oil volume was read 30 min after heating was stopped. The collected oil was transferred into glass vials, dried with anhydrous sodium sulfate, and then stored at −20 °C.

Essential oils obtained from both the primary plant material and the post-extraction material were subjected to qualitative and quantitative chromatographic analysis (GC-FID, GC-MS).

### 3.8. Indirect Estimation of EO Recovery in Infusions (Depletion-Based Approach)

The concentration of essential oil (EO) in the herbal extracts was determined using an indirect approach, based on the difference in the content of volatile constituents between the primary plant material and the post-extraction material. This calculation was performed under the assumption that no losses of volatile substances occurred during the extraction process and with consideration of the density of the essential oil [47].

### 3.9. Chromatographic Analysis

Chromatographic analyses were performed using gas chromatography coupled with a flame ionization detector (GC-FID) and a mass spectrometer (GC-MS), according to the procedure described by Kowalski and Wawrzykowski [110].

The GC-MS system consisted of a Varian GC-3800 gas chromatograph (Varian, Walnut Creek, CA, USA) coupled with a Varian 4000 MS/MS mass detector (Varian, Walnut Creek, CA, USA), equipped with a VF-5MS capillary column with a stationary phase composed of 5% phenyl and 95% dimethylpolysiloxane. The following temperature program was applied: 0.5 min at 60 °C, followed by an increase to 246 °C at a rate of 3 °C/min, and holding at 246 °C for 10 min. The injector temperature was set at 250 °C, and the transfer line temperature was also 250 °C. The split ratio was 1:50. Helium was used as the carrier gas at a constant volumetric flow rate of 0.5 mL/min. Ionization was performed using an electron impact source with an ionization energy of 70 eV, and mass spectra were recorded over a mass range of 40–1000 Da. The injection volume was 1 µL.

The GC-FID system consisted of a Varian GC-3800 gas chromatograph (Varian, Walnut Creek, CA, USA) coupled with an FID detector and equipped with a DB-5 capillary column (stationary phase: 95% dimethylpolysiloxane and 5% phenyl). Chromatographic separation conditions were identical to those applied for the GC-MS analysis.

Qualitative analysis was performed by comparing the retention indices of the analyzed compounds with literature data, as well as by matching the obtained mass spectra of separated volatile constituents with reference spectra stored in the applied libraries [97,98,111].

Quantitative analysis of the essential oil was carried out using the internal normalization method, determining the relative contribution of individual essential oil components to the total content of all identified volatile compounds.

### 3.10. Extraction Yield

The extraction yield for total phenolic content (TPC) and total flavonoid content (TFC) was calculated as the percentage share of biologically active compounds present in a given extract relative to their content in the reference methanolic extract [78]. Methanol was treated as the reference solvent, as it was assumed to enable optimal extraction of active constituents. Therefore, the concentration of these compounds obtained in methanol was considered to represent 100% extraction efficiency. The extraction yield obtained using the solvents applied in this study was calculated according to Equation (1):

(1) $$W\;[\%]\;=\;\frac{C_{Sol}*100\%}{C_{Met}}$$

where

*C_Met_*—concentration of the compound (TPC, TFC) in the extract [mg/L] obtained using methanol (reference solvent—methanolic extraction);

*C_Sol_*—concentration of the compound (TPC, TFC) [mg/L] in the extract obtained using the selected extraction system.

Essential oil (EO): The extraction yield for EO was determined as the recovery of the volatile fraction from the plant material relative to the total EO content in the initial plant material, according to Equation (2):

(2) $$W\;[\%]\;=\;\frac{{(Z}_{0}\;-\;Z_{PE})*100\%}{Z_{0}}$$

where

*Z*_0_—EO content in the initial plant raw material, determined by distillation [% *v*/*w*];

*Z_PE_*—EO content in the post-extraction plant material, determined by distillation [% *v*/*w*].

### 3.11. Relative Contribution of Active Compounds in Primary and Secondary Extracts

The relative percentage contribution of the analyzed active substances in the total fraction of primary and secondary extracts was calculated assuming that the combined content of TPC or TFC in both extracts represents 100% (Equation (3)).

(3) $$W\;[\%]=\frac{C_{p\;}*100\%}{{C_{p}+C}_{w}}$$

where

*C_p_*—concentration of individual biologically active compounds (TPC, TFC) in extracts obtained using an initial extraction temperature of 100 °C and an extraction time of 10 min (primary extraction);

*C_w_*—concentration of individual biologically active compounds (TPC, TFC) in extracts obtained using an extraction temperature of 100 °C and an extraction time of 10 min (secondary extraction).

### 3.12. Free Radical-Scavenging Ability by the Use of a Stable DPPH^•^ Radical

Antioxidant activity was evaluated using the DPPH radical scavenging assay, based on the method originally proposed by Brand-Williams et al., with minor modifications [46,112]. The synthetic radical 2,2-diphenyl-1-picrylhydrazyl (DPPH; Sigma) was dissolved in ethanol, and absorbance measurements were performed at 517 nm. The antioxidant activity determined by this assay was expressed as Trolox equivalents (TE). Prior to analysis, the DPPH solution was adjusted to obtain an initial absorbance of approximately 0.9 at 517 nm and was protected from light.

For the reaction, 1.5 mL of the DPPH solution was mixed with 20 µL of the tested extracts. The decrease in absorbance (A) was recorded after 30 min of incubation. The percentage of DPPH radical inhibition was calculated according to Equation (4):

(4) $$\mathrm{inhibition}\;[\%]\;=\;\frac{100*({A_{0}\;-\;A}_{1})}{A_{0}}$$

where

A_0_—absorbance of the control sample;

A_1_—absorbance of the tested sample.

The antioxidant capacity was expressed as Trolox equivalents (TE), based on linear calibration curves constructed using nine Trolox standard solutions in the concentration range of 0.2–2.0 mM, following the procedure described by Rumpf et al. [113]. Prior to analysis, each sample was appropriately diluted to ensure that absorbance values fell within the linear range of the calibration curve. All determinations were performed in triplicate.

### 3.13. Ferric Reducing Antioxidant Power (FRAP) Assay

The antioxidant capacity of the samples was evaluated using the ferric reducing antioxidant power (FRAP) assay, which measures the ability of antioxidants to reduce Fe(III) to Fe(II) [114]. The FRAP working reagent was freshly prepared by combining acetate buffer (300 mM, pH 3.6), a 10 mM solution of TPTZ in 40 mM HCl, and 20 mM FeCl_3_ in a volumetric ratio of 10:1:1 (*v*/*v*/*v*).

For the assay, 3000 µL of the FRAP reagent was mixed with 100 µL of the tested extract, and the mixture was thoroughly homogenized. After incubation, absorbance was recorded at 593 nm following 180 min of reaction. The final antioxidant activity values were expressed as Trolox equivalents (TE). Calibration of the assay was initially performed using iron(II) sulfate solutions within the concentration range of 100–1000 µM. All reagents and solutions were prepared fresh and used on the day of analysis. One FRAP unit corresponds to the reducing capacity equivalent to 1 mM of Fe(III) converted to Fe(II).

Antioxidant activity was finally expressed as Trolox equivalents (TE), based on linear calibration curves constructed from nine Trolox standard solutions with concentrations ranging from 0.05 to 0.5 mM in the FRAP assay, according to the procedure described by Rumpf et al. [113]. Prior to measurement, samples were appropriately diluted to ensure that the absorbance values fell within the linear range of the Trolox calibration curve. All measurements were performed in triplicate for each sample.

### 3.14. Statistical Analysis

Statistical evaluation of the results was performed using one-way analysis of variance (ANOVA), followed by Duncan’s multiple range test, implemented in the SAS statistical software package (version 9.1; SAS Institute, Cary, NC, USA). Differences were considered statistically significant at *p* < 0.05. The results are presented as mean values from three independent replicates; standard deviation (SD) is reported either directly, in table footnotes, or graphically as error bars, depending on the format of data presentation. Values marked with different letters (a, b, c, etc.) indicate statistically significant differences between samples at *p* < 0.05.

## 4. Summary

The study showed that low-processed, infusion-like extraction of peppermint leaves can be precisely controlled through the selection of food-grade solvents differing in polarity, viscosity, and phase affinity. Under short, hot extraction conditions, water and nanowater provided the highest relative recovery of polar antioxidants (TPC/TFC) when yields were expressed relative to an exhaustive 70% methanolic Soxhlet extract (taken as 100% for TPC/TFC). In contrast, the ethanol–water mixture (50%) and rapeseed oil preferentially extracted the volatile and lipophilic fraction, as reflected by essential oil recovery. Importantly, the post-extraction biomass remained a significant source of bioactive compounds, and secondary extraction often contributed a substantial share of the combined TPC/TFC, confirming the feasibility of simple utilization of plant residues within a circular-economy model. In general, a sequential solvent strategy is recommended (aqueous or glycerol–water systems for TPC/TFC recovery followed by ethanol–water or edible oil for EO recovery) in order to obtain extracts intended for specific end-use applications while maintaining mild processing conditions and compliance with green chemistry principles.

## 5. Conclusions

- Under short, infusion-like extraction conditions, solvent selection strongly determines process selectivity: aqueous media favor recovery of polar phytochemicals (TPC/TFC), whereas the ethanol–water mixture (50%) and rapeseed oil favor recovery of essential oil constituents.
- The highest TPC/TFC yields under the selected “optimal conditions” were obtained for water and nanowater, which also showed the strongest antioxidant activity (DPPH/FRAP), consistent with the distribution of phenolic antioxidants in peppermint leaves.
- The ethanol–water mixture (50%) and rapeseed oil exhibited the highest EO recovery, and the two-step extraction scheme enabled near-complete depletion of EO in the ethanol system, indicating high phase affinity toward peppermint volatiles.
- Secondary extraction allowed recovery of a substantial additional fraction of bioactive compounds, often exceeding 30% of the combined TPC/TFC across solvent systems, confirming that post-extraction residues represent a valuable raw material rather than waste.
- From an application perspective, a sequential extraction strategy is recommended: (i) water/nanowater or a glycerol–water system to obtain phenolic-rich extracts with high antioxidant capacity, followed by (ii) ethanol–water or edible oil to obtain essential-oil-enriched preparations.
- From a practical perspective, this strategy may support the design of application-oriented peppermint preparations obtained with food-grade solvents, including phenolic-rich antioxidant extracts and essential-oil-enriched formulations, while simultaneously reducing waste through secondary use of post-extraction biomass.
- Overall, the proposed low-processed, food-grade approach represents a practical pathway for sustainable production of peppermint extracts and straightforward utilization of post-extraction biomass within a circular-economy framework.

## 6. Limitations

The present study has several limitations that should be considered when interpreting the results. First, the peppermint leaves originated from a single commercial batch and one particle-size fraction; therefore, seasonal, cultivar-related, and agronomic variability were not evaluated. Second, the main extraction protocol was intentionally designed as a short, infusion-like process using food-grade solvents; consequently, the reported yields reflect recovery obtained under mild, low-processed conditions. It should be emphasized that the definition of TPC/TFC yield is relative: a 70% methanolic Soxhlet extract (12 h) was treated as an exhaustive extract and set to 100% according to a previously reported methodological approach [78]. As a result, the percentages reported for infusion-like extractions quantify the degree of recovery relative to a reference extract rather than representing absolute extraction “strength” across fundamentally different extraction modes (short batch extraction versus continuous Soxhlet extraction). Third, phenolic composition was assessed using global indices (TPC/TFC) rather than targeted chromatographic quantification of individual phenolic compounds; similarly, EO extraction performance was largely inferred from depletion/recovery metrics in plant material before and after extraction rather than from direct quantification of volatile concentrations in each liquid extract across all solvent systems. Finally, storage stability, microbiological safety of food-grade extracts, sensory acceptance, and process scale-up (including energy and economic assessment) were outside the scope of the present study and should be addressed in future work.

## Figures and Tables

**Table 1:** Comparison of polyphenol and flavonoid concentrations in 65% and 50% glycerol–water extracts (initial extraction temperature 100 °C; extraction time 10 min). Different letters (a) indicate statistically significant differences at *p* < 0.05.

Extraction System	Concentration ± SD [mg/L]	
	TPC	TFC
Glycerol (65%, 100 °C)	516.33 ^a^ ± 35.02	397.62 ^a^ ± 16.29
Glycerol (50%, 100 °C)	520.36 ^a^ ± 27.79	401.25 ^a^ ± 18.74

**Table 2:** Percentage content [% *v*/*w*] of EO in peppermint leaves after primary and secondary extraction.

EO Content in Plant Material [% *v*/*w*]																
Raw Material	Water 100 °C 10 min	Water 100 °C 6 min	Water 80 °C 10 min	Water 80 °C 6 min	Glycerol 65% 100 °C 10 min	Glycerol 65% 100 °C 6 min	Glycerol 65% 80 °C 10 min	Glycerol 65% 80 °C 6 min	Rapeseed Oil 100 °C 10 min	Rapeseed Oil 100 °C 6 min	Rapeseed Oil 80 °C 10 min	Rapeseed Oil 80 °C 6 min	Ethanol 50%, RT 10 min	Ethanol 50%, RT 6 min	Ethanol 50%, 50 °C 10 min	Ethanol 50%, 50 °C 6 min
after primary extraction																
1.40 ^a^	1.11 ^d^	1.26 ^c^	1.35 ^b^	1.35 ^b^	1.03 ^f^	1.15 ^d^	1.08 ^e^	1.10 ^e^	0.50 ^j^	0.73 ^g^	0.65 ^h^	0.65 ^h^	0.50 ^j^	0.55 ^i^	0.45 ^k^	0.45 ^k^
after secondary extraction																
1.40 ^a^	0.45 ^g^	0.45 ^g^	0.50 ^f^	0.55 ^ef^	0.70 ^c^	0.85 ^b^	0.60 ^d^	0.65 ^cd^	0.05 ^h^	0.05 ^h^	0.02 ^i^	0.01 ^i^	0.00 ^i^	0.00 ^i^	0.00 ^i^	0.00 ^i^

Different letters (a, b, c, etc.) show a significant difference with *p* < 0.05. Values are presented as means rounded to two decimal places; therefore, some closely similar results may appear numerically identical in the table. The first column represents the initial raw material and serves as a reference value.

**Table 3:** EO remaining in plant materials after primary and secondary extraction, expressed as relative percentage contribution.

EO Remaining After Primary Extraction [%]															
Water 100 °C 10 min	Water 100 °C 6 min	Water 80 °C 10 min	Water 80 °C 6 min	Glycerol 65% 100 °C 10 min	Glycerol 65% 100 °C 6 min	Glycerol 65% 80 °C 10 min	Glycerol 65% 80 °C 6 min	Rapeseed Oil 100 °C 10 min	Rapeseed Oil 100 °C 6 min	Rapeseed Oil 80 °C 10 min	Rapeseed Oil 80 °C 6 min	Ethanol 50%, RT 10 min	Ethanol 50%, RT 6 min	Ethanol 50%, 50 °C 10 min	Ethanol 50%, 50 °C 6 min
after primary extraction															
79.29 ^cd^	90.00 ^b^	96.43 ^a^	96.43 ^a^	73.57 ^e^	82.14 ^c^	77.14 ^d^	78.57 ^d^	35.71 ^i^	52.14 ^f^	46.43 ^g^	46.43 ^g^	35.71 ^i^	39.29 ^h^	32.14 ^j^	32.14 ^j^
after secondary extraction															
32.14 ^f^	32.14 ^f^	35.71 ^e^	39.28 ^de^	50.00 ^b^	60.71 ^a^	42.86 ^d^	46.43 ^c^	3.57 ^g^	3.57 ^g^	1.43 ^h^	0.71 ^i^	0.00 ^j^	0.00 ^j^	0.00 ^j^	0.00 ^j^

Different letters (a, b, c, etc.) show a significant difference with *p* < 0.05. Values are presented as means rounded to two decimal places; therefore, some closely similar results may appear numerically identical in the table. The first column corresponds to the reference extraction condition (water, 100 °C, 10 min).

**Table 4:** EO released during primary and secondary extraction from plant materials, expressed as relative percentage contribution.

EO Released [%]															
Water 100 °C 10 min	Water 100 °C 6 min	Water 80 °C 10 min	Water 80 °C 6 min	Glycerol 65% 100 °C 10 min	Glycerol 65% 100 °C 6 min	Glycerol 65% 80 °C 10 min	Glycerol 65% 80 °C 6 min	Rapeseed Oil 100 °C 10 min	Rapeseed Oil 100 °C 6 min	Rapeseed Oil 80 °C 10 min	Rapeseed Oil 80 °C 6 min	Ethanol 50%, RT 10 min	Ethanol 50%, RT 6 min	Ethanol 50%, 50 °C 10 min	Ethanol 50%, 50 °C 6 min
after primary extraction															
20.71 ^h^	10.00 ^j^	3.57 ^k^	3.57 ^k^	26.43 ^f^	17.86 ^i^	22.86 ^gh^	21.43 ^gh^	64.29 ^b^	47.86 ^e^	53.57 ^d^	53.57 ^d^	64.29 ^b^	60.71 ^c^	67.86 ^a^	67.86 ^a^
after secondary extraction															
67.86 ^c^	67.86 ^c^	64.29 ^d^	60.72 ^e^	50.00 ^h^	39.29 ^i^	57.14 ^f^	53.57 ^g^	96.43 ^b^	96.43 ^b^	98.57 ^a^	99.29 ^a^	100.00 ^a^	100.00 ^a^	100.00 ^a^	100.00 ^a^

Different letters (a, b, c, etc.) show a significant difference with *p* < 0.05. Values are presented as means rounded to two decimal places; therefore, some closely similar results may appear numerically identical in the table. The first column corresponds to the reference extraction condition (water, 100 °C, 10 min).

**Table 5:** Composition of EO isolated from peppermint.

Compound	RI	RI_LIT_ [97]	Relative Content ± SD		
			[%]		
α-Pinene	927	932	0.47	±	0.02
Thuja-2,4(10)-diene	947	953	0.03	±	0.00
Sabinene	966	969	0.37	±	0.02
β-Pinene	972	974	0.77	±	0.03
Myrcene	984	988	0.20	±	0.01
3-Octanol	992	988	0.21	±	0.02
p-Cymene	1018	1020	0.04	±	0.00
Limonene	1022	1024	0.45	±	0.02
1,8-Cineole	1025	1026	3.07	±	0.25
Z-β-Ocimene	1028	1032	0.12	±	0.02
E-β-Ocimene	1038	1044	tr.	±	
γ-Terpinene	1049	1054	0.02	±	0.00
cis-Sabinene hydrate	1060	1065	0.04	±	0.00
Terpinolene	1074	1086	tr.	±	
Linalool	1087	1095	0.29	±	0.02
Isopentyl isovalerate	1094	1102	0.03	±	0.00
Octyl octanoate	1106	1120	0.03	±	0.00
trans-Sabinol	1130	1137	0.21	±	0.02
cis-Verbenol	1132	1137	0.06	±	0.00
trans-Verbenol	1136	1140	0.11	±	0.01
Isopulegol	1139	1145	0.17	±	0.01
Menthone	1148	1148	32.06	±	0.93
Isomenthone	1157	1158	6.32	±	0.52
Neomenthol	1163	1161	2.19	±	0.18
Menthol	1173	1167	36.1	±	0.85
Isomenthol	1183	1179	0.48	±	0.03
Neo-isomenthol	1187	1184	0.16	±	0.01
Myrtenol	1191	1194	0.14	±	0.01
trans-Carveol	1205	1215	0.06	±	0.00
Pulegone	1236	1233	1.72	±	0.08
Carvone	1243	1239	0.02	±	0.00
Piperitone	1253	1249	1.46	±	0.07
trans-Myrtanol	1263	1258	0.02	±	0.00
Neomenthyl acetate	1271	1271	0.19	±	0.01
Limonen-10-ol	1276	1288	0.03	±	0.00
Menthyl acetate	1290	1294	10.32	±	0.09
Isomenthyl acetate	1305	1304	0.23	±	0.02
Neo-isopulegyl acetate	1308	1312	0.03	±	0.00
Eugenol	1351	1356	0.04	±	0.00
α-Copaene	1372	1374	0.06	±	0.00
β-Bourbonene	1379	1387	0.57	±	0.02
β-Elemene	1385	1389	0.78	±	0.03
E-Jasmone	1390	1390	0.05	±	0.00
cis-α-Bergamotene	1398	1411	0.08	±	0.00
Sum			99.80%		

RI—retention indices (from temperature programming, using definition of Van den Dool and Kratz [98]); RI_LIT_—retention indices from literature [97]; tr.—less than 0.01%.

**Table 6:** Percentage contribution of main components in EO isolated from peppermint after primary extraction.

Percentage																	
Compound	Raw Material	Water 100 °C 10 min	Water 100 °C 6 min	Water 80 °C 10 min	Water 80 °C 6 min	Glycerol 65% 100 °C 10 min	Glycerol 65% 100 °C 6 min	Glycerol 65% 80 °C 10 min	Glycerol 65% 80 °C 6 min	Rapeseed Oil 100 °C 10 min	Rapeseed Oil 100 °C 6 min	Rapeseed Oil 80 °C 10 min	Rapeseed Oil 80 °C 6 min	Ethanol 50%, RT 10 min	Ethanol 50%, RT 6 min	Ethanol 50%, 50 °C 10 min	Ethanol 50%, 50 °C 6 min
Limonene	0.45 ^a^	0.37 ^d^	0.34 ^g^	0.22 ^k^	0.35 ^f^	0.34 ^g^	0.40 ^b^	0.39 ^c^	0.37 ^d^	0.18 ^m^	0.21 ^L^	0.15 ^o^	0.16 ^n^	0.25 ^j^	0.36 ^e^	0.29 ^h^	0.27 ^i^
1,8-Cineole	3.07 ^b^	2.02 ^f^	1.74 ^h^	1.53 ^i^	2.56 ^e^	1.79 ^h^	2.05 ^f^	2.69 ^d^	2.59 ^e^	1.36 ^j^	1.52 ^i^	1.26 ^k^	1.29 ^k^	1.93 ^g^	3.44 ^a^	2.89 ^c^	2.56 ^e^
Linalool	0.29 ^d^	0.24 ^e^	0.19 ^i^	0.21 ^g^	0.31 ^c^	0.32 ^b^	0.38 ^a^	0.23 ^f^	0.21 ^g^	0.23 ^f^	0.24 ^e^	0.1 ^L^	0.12 ^k^	0.12 ^k^	0.20 ^h^	0.19 ^i^	0.17 ^j^
Menthone	36.1 ^a^	25.89 ^de^	22.41 ^i^	24.25 ^g^	25.03 ^f^	22.81 ^hi^	21.51 ^j^	19.83 ^k^	20.05 ^k^	25.46 ^ef^	26.28 ^d^	24.3 ^g^	23.18 ^h^	22.14 ^ij^	32.06 ^b^	30.25 ^c^	31.56 ^b^
Isomenthone	6.32 ^e^	5.92 ^g^	5.76 ^h^	6.5 ^d^	6.97 ^c^	6.01 ^fg^	4.43 ^i^	6.32 ^e^	6.13 ^f^	7.10 ^b^	7.87 ^a^	2.81 ^j^	2.68 ^k^	4.37 ^i^	7.19 ^b^	6.14 ^f^	5.89 ^g^
Neomenthol	2.19 ^h^	2.81 ^c^	2.56 ^e^	2.87 ^b^	2.66 ^d^	2.48 ^f^	2.31 ^g^	2.69 ^d^	2.58 ^e^	2.77 ^c^	2.93 ^a^	0.88 ^L^	0.45 ^m^	0.35 ^n^	1.89 ^i^	1.56 ^j^	1.48 ^k^
Menthol	22.45 ^h^	31.46 ^a^	26.92 ^d^	31.12 ^a^	26.88 ^d^	24.45 ^e^	22.89 ^gh^	23.15 ^g^	22.89 ^gh^	28.39 ^c^	29.66 ^b^	9.03 ^k^	7.72 ^L^	1.71 ^m^	23.77 ^f^	20.69 ^i^	19.56 ^j^
Isomenthol	0.33 ^L^	0.52 ^i^	0.60 ^f^	0.68 ^c^	0.59 ^g^	0.57 ^h^	0.52 ^i^	0.49 ^j^	0.48 ^k^	0.64 ^e^	0.65 ^d^	0.81 ^a^	0.79 ^b^	0.26 ^n^	0.26 ^n^	0.28 ^m^	0.25 ^o^
Pulegone	1.72 ^c^	0.24 ^ghi^	0.25 ^fgh^	0.26 ^fgh^	0.28 ^ef^	0.26 ^fgh^	0.45 ^d^	2.22 ^a^	1.80 ^b^	0.31 ^e^	0.31 ^e^	0.21 ^ij^	0.27 ^fg^	0.18 ^j^	0.23 ^hi^	0.19 ^j^	0.14 ^k^
piperitone	1.46 ^c^	1.24 ^f^	1.08 ^h^	1.34 ^e^	1.35 ^e^	1.12 ^g^	0.98 ^i^	0.90 ^j^	0.85 ^k^	1.51 ^b^	1.56 ^a^	0.60 ^n^	0.69 ^m^	0.77 ^L^	1.58 ^a^	1.47 ^c^	1.38 ^d^
Menthyl acetate	10.32 ^f^	10.12 ^f^	12.88 ^b^	13.69 ^a^	9.73 ^g^	12.56 ^c^	12.56 ^c^	11.59 ^d^	10.57 ^e^	13.77 ^e^	11.39 ^d^	3.58 ^k^	1.93 ^L^	6.66 ^i^	6.98 ^h^	5.98 ^j^	6.12 ^j^

Different letters (a, b, c, etc.) show a significant difference with *p* < 0.05.

**Table 7:** Percentage contribution of main components in EO isolated from peppermint after secondary extraction.

Percentage																	
Compound	Raw Material	Water 100 °C 10 min	Water 100 °C 6 min	Water 80 °C 10 min	Water 80 °C 6 min	Glycerol 65% 100 °C 10 min	Glycerol 65% 100 °C 6 min	Glycerol 65% 80 °C 10 min	Glycerol 65% 80 °C 6 min	Rapeseed Oil 100 °C 10 min	Rapeseed Oil 100 °C 6 min	Rapeseed Oil 80 °C 10 min	Rapeseed Oil 80 °C 6 min	Ethanol 50%, RT 10 min	Ethanol 50%, RT 6 min	Ethanol 50%, 50 °C 10 min	Ethanol 50%, 50 °C 6 min
Limonene	0.45 ^b^	0.23 ^g^	0.25 ^f^	0.19 ^i^	0.21 ^h^	0.42 ^c^	0.38 ^e^	0.41 ^d^	0.5 ^a^	0.09 ^j^	0.02 ^p^	0.06 ^m^	0.04 ^n^	0.03 ^o^	0.06 ^m^	0.07 ^L^	0.08 ^k^
1,8-Cineole	3.07 ^a^	1.52 ^a^	1.53 ^e^	1.45 ^f^	1.38 ^g^	2.05 ^c^	1.86 ^d^	2.12 ^b^	1.53 ^e^	0.67 ^k^	0.58 ^L^	0.61 ^kL^	0.63 ^kL^	0.19 ^m^	1.05 ^i^	0.9 ^j^	1.12 ^h^
Linalool	0.29 ^c^	0.14 ^h^	0.18 ^e^	0.17 ^f^	0.19 ^d^	1.04 ^b^	1.2 ^a^	0.17 ^f^	0.15 ^g^	0.09 ^i^	0.05 ^m^	0.07 ^k^	0.06 ^L^	0.08 ^j^	0.06 ^L^	0.05 ^m^	0.06 ^L^
Menthone	36.1 ^a^	19.8 ^b^	18.86 ^c^	17.4 ^d^	17.5 ^d^	11.85 ^g^	13.85 ^ef^	14.57 ^e^	13.5 ^f^	10.56 ^h^	10.62 ^h^	11.8 ^g^	12.1 ^g^	12.5 ^g^	14.2 ^ef^	18.5 ^c^	19.25 ^bc^
Isomenthone	6.32 ^a^	4.52 ^d^	3.89 ^f^	4.58 ^d^	3.45 ^g^	3.12 ^h^	2.85 ^i^	3.17 ^h^	2.27 ^k^	2.56 ^j^	1.85 ^m^	2.13 ^L^	1.91 ^m^	3.14 ^h^	4.16 ^e^	5.17 ^b^	4.89 ^c^
Neomenthol	2.19 ^a^	1.56 ^c^	1.62 ^b^	1.32 ^d^	1.21 ^e^	0.85 ^h^	0.74 ^i^	0.89 ^h^	0.62 ^j^	1.03 ^f^	1.02 ^f^	0.97 ^g^	0.85 ^h^	0.15 ^k^	0.18 ^k^	1.05 ^f^	0.75 ^i^
Menthol	22.45 ^b^	23.46 ^a^	20.86 ^c^	19.86 ^d^	17.6 ^e^	7.45 ^j^	8.95 ^i^	9.12 ^i^	6.8 ^k^	15.68 ^f^	13.15 ^h^	14.2 ^g^	13.8 ^g^	0.89 ^n^	5.26 ^m^	6.14 ^L^	7.12 ^jk^
Isomenthol	0.33 ^c^	0.26 ^f^	2.27 ^a^	0.12 ^m^	1.02 ^b^	0.13 ^L^	0.26 ^f^	0.24 ^g^	0.28 ^e^	0.31 ^d^	0.21 ^h^	0.24 ^g^	0.26 ^f^	0.14 ^k^	0.17 ^i^	0.18 ^j^	0.14 ^k^
Pulegone	1.72 ^b^	0.16 ^e^	0.17 ^e^	0.16 ^e^	0.19 ^e^	1.46 ^d^	1.75 ^b^	1.67 ^c^	1.8 ^a^	0.05 ^fg^	0.03 ^gh^	0.03 ^gh^	0.01 ^h^	0.06 ^fg^	0.18 ^e^	0.05 ^fg^	0.07 ^f^
piperitone	1.46 ^b^	0.97 ^f^	0.87 ^g^	0.79 ^h^	0.74 ^i^	1.52 ^a^	1.32 ^d^	1.12 ^e^	1.37 ^c^	0.89 ^g^	0.58 ^k^	0.86 ^g^	0.67 ^j^	0.21 ^o^	0.34 ^n^	0.41 ^m^	0.38 ^L^
Menthyl acetate	10.32 ^a^	3.17 ^f^	2.85 ^g^	4.12 ^c^	3.85 ^d^	3.12 ^f^	3.51 ^e^	3.10 ^f^	2.31 ^h^	2.12 ^hi^	1.92 ^ij^	2.02 ^ij^	1.85 ^j^	2.02 ^ij^	3.15 ^f^	4.12 ^c^	5.18 ^b^

Different letters (a, b, c, etc.) show a significant difference with *p* < 0.05.

**Table 8:** Concentration, time, and temperature variants applied in the extraction procedures.

Solvent	Initial Extraction Temperature	Extraction Time
Aqueous glycerol solution (65% and 50%)	50 °C, 80 °C, 100 °C	2–30 min
Distilled water		
Nanowater		
Rapeseed oil		
Aqueous ethanol solution (50%)	50 °C, room temperature	

## Data Availability

Data are contained within the article.

## Abbreviations

The following abbreviations are used in this manuscript:

TPC	total phenolic content
TFC	total flavonoid content
EO	essential oil
DPPH	2,2-diphenyl-1-picrylhydrazyl
FRAP	ferric reducing antioxidant power
GC-FID	gas chromatography–flame ionization detection
GC-MS	gas chromatography–mass spectrometry
